# High performing and stable supported nano-alloys for the catalytic hydrogenation of levulinic acid to γ-valerolactone

**DOI:** 10.1038/ncomms7540

**Published:** 2015-03-17

**Authors:** Wenhao Luo, Meenakshisundaram Sankar, Andrew M. Beale, Qian He, Christopher J. Kiely, Pieter C. A. Bruijnincx, Bert M. Weckhuysen

**Affiliations:** 1 Inorganic Chemistry and Catalysis, Debye Institute for Nanomaterials Science, Utrecht University, Universiteitsweg 99, 3584 CG Utrecht, The Netherlands; 2 UK Catalysis Hub, Research Complex at Harwell, Rutherford Appleton Laboratory, Oxfordshire OX11 0QX, UK; 3 Department of Chemistry, University College London, 20 Gordon Street, London WC1H 0AJ, UK; 4 Department of Materials Science and Engineering, Lehigh University, 5 East Packer Avenue, Bethlehem, Pennsylvania 18015, USA

## Abstract

The catalytic hydrogenation of levulinic acid, a key platform molecule in many biorefinery schemes, into γ-valerolactone is considered as one of the pivotal reactions to convert lignocellulose-based biomass into renewable fuels and chemicals. Here we report on the development of highly active, selective and stable supported metal catalysts for this reaction and on the beneficial effects of metal nano-alloying. Bimetallic random alloys of gold-palladium and ruthenium-palladium supported on titanium dioxide are prepared with a modified metal impregnation method. Gold-palladium/titanium dioxide shows a marked,~27-fold increase in activity (that is, turnover frequency of 0.1 s^−1^) compared with its monometallic counterparts. Although ruthenium-palladium/titanium dioxide is not only exceptionally active (that is, turnover frequency of 0.6 s^−1^), it shows excellent, sustained selectivity to γ-valerolactone (99%). The dilution and isolation of ruthenium by palladium is thought to be responsible for this superior catalytic performance. Alloying, furthermore, greatly improves the stability of both supported nano-alloy catalysts.

## Abstract (web summary)

The hydrogenation of leuvinic acid to γ-valerolactone is an important step in the conversion of lignocellulose to high value chemicals. Here, the authors report that bimetallic alloys are active and stable catalysts for this reaction, and attribute this to geometric and electronic effects.

## Graphical abstract

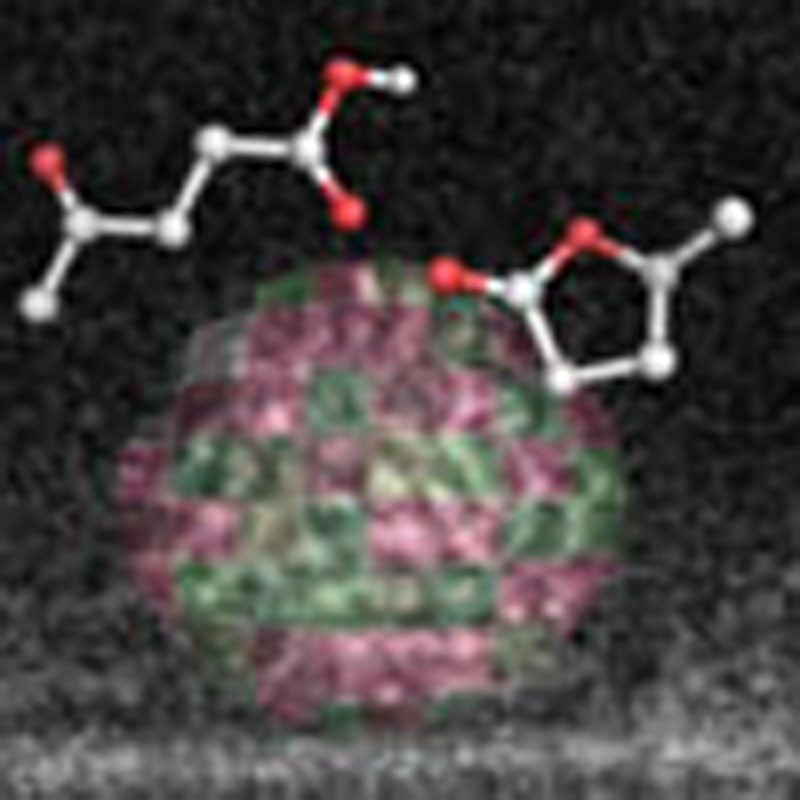

Various biomass feedstocks and many catalytic conversion routes, involving numerous intermediates and end products, are currently being explored for the production of renewable chemicals and fuels in biorefinery-type operations. A small number of compounds have emerged, however, with the potential to play a pivotal role as primary biorefinery building blocks, that is, as so-called renewable platform molecules^1^. Levulinic acid (LA) is one of these promising platform molecules, as it is produced easily and economically from the carbohydrates in lignocellulosic biomass^2^,^3^,^4^. Many value-added products can in turn be obtained from LA, including polymer monomers (for example, succinic and adipic acid), solvents (for example, methyltetrahydrofuran (MTHF))^5^, plasticizers (for example, 1,4-pentanediol (PD))^5^, and fuel components and their precursors (for example, butenes^6^, valeric acid esters^7^ and nonanone^8^). Many of these routes actually involve γ-valerolactone (GVL), a versatile platform molecule in its own right^9^, as intermediate, making the hydrogenation of LA to GVL a reaction of immense importance (Fig. 1)^6^,^7^,^10^.

Many metal-based catalysts have been reported for LA hydrogenation and the application of heterogeneous catalysts, which should in principle be most suitable for large-scale GVL manufacturing, in this reaction has been recently reviewed^10^. Supplementary Table 1 provides an overview of some selected catalyst materials, including productivity values and reaction conditions, reported for the LA-to-GVL process. Supported monometallic noble metal catalysts have been studied most and are typically used with molecular hydrogen in the liquid phase at temperatures ranging from 298 to 523 K. Ru-based catalysts generally give the highest GVL productivities, with Ru/C being highly active and selective for hydrogenation of LA. While carbon supports are stable under the highly polar and acidic conditions of a LA hydrogenation process, they do not survive the multiple regeneration cycles required for catalyst reactivation by burning-off coke at high temperatures^7^. In this respect, metal oxide supports, such as TiO_2_, provide a promising alternative on account of their good stability under both reaction and regeneration conditions^7^,^11^,^12^.

Supported bimetallic heterogeneous catalysts have been widely used in many organic transformations, as such combinations of metals in appropriate proportions and nanostructures can often lead to superior catalytic performance^13^. The advantages that bimetallic catalysts might bring have not yet been fully exploited for biomass conversion reactions, however, especially not for the conversion of LA to GVL^10^,^14^. Indeed, the few reported examples have actually met with mixed success. Braden *et al*.^15^ showed that alloying Ru with Re improved catalyst stability against traces of sulfuric acid in the LA feed, traces which would rapidly deactivate the monometallic Ru/C catalyst. The activity of the bimetallic catalyst was considerably lower than the activity of Ru/C in the absence of sulfuric acid, however. Wettstein *et al*.^16^ reported that alloying Sn with Ru/C led to highly stable and GVL-selective catalysts in the hydrogenation of LA, but again at the expense of activity. In contrast, Lange *et al*.^11^ concluded that alloying of supported Pt catalysts with Re or Ru did not improve performance, but led to reduced initial activity and enhanced deactivation instead.

Very recently, Yang *et al*.^17^ reported on the use of Ru−Ni bimetallic nanoparticles inside ordered mesoporous carbons; the bimetallic catalyst proved highly stable over multiple reuse cycles displaying an activity that is similar to the corresponding monometallic Ru catalyst. In another recent report, Shimizu *et al*.^18^ developed Ni-MoO_x_-based catalysts as a non-noble metal-based alternative to Ru/TiO_2_. A beneficial effect of alloying on activity was suggested, but the catalysts were not quite as active as the benchmark Ru-based ones.

As with monometallic catalysts, the performance of supported bimetallic nanoparticles is highly dependent on their compositional and structural characteristics, features that are ultimately determined by the preparation method^19^. Although many synthesis strategies have been reported, precise control over both particle size and the extent of alloy mixing is still a challenge. Recently, one of us reported on an ‘excess-anion’, modified impregnation methodology (*M*_Im_) for the synthesis of supported Au-Pd catalysts with very narrow particle size distributions and a quite homogeneous, random alloy structure^20^. The *M*_Im_ procedure involves the addition of an excess of chloride ions (in the form of dilute HCl) in the impregnation solution during the preparation of supported Au-Pd nanoparticles. This excess of chloride ions eliminates one of the major structural problems observed in supported Au-Pd nanoparticles, that is, size-dependent compositional variation. In other words, this excess chloride ions increases the concentration of Au in the small Au-Pd particles at the expense of large micrometre-sized gold-only particles^20^. The Au-Pd catalysts were found to be very active in direct hydrogen peroxide synthesis and aerobic alcohol oxidation^21^.

In this work, we demonstrate that the controlled preparation of supported bimetallic nanoparticles via the *M*_Im_ synthesis methodology has a marked, positive effect on the catalysts’ activity, selectivity and stability in the hydrogenation of LA to GVL. The performance of the bimetallic catalysts Au-Pd/TiO_2_ and Ru-Pd/TiO_2_ (both with equimolar metal ratio) is compared with their monometallic counterparts. Insights into the reasons for the superior catalytic performance of bimetallic systems were provided by a combination of scanning transmission electron microscopy (STEM), X-ray absorption spectroscopy (XAS), X-ray photoelectron spectroscopy (XPS) and Fourier transform infrared spectroscopy (FT-IR) studies using CO as a probe molecule. On the basis of this detailed characterization, it is found that the beneficial effect of proper Au-Pd and Ru-Pd nano-alloying is the result of electronic modification of Pd in the former catalyst and dilution and isolation of Ru in the latter.

## Results

### Catalyst performance and stability

**Figure 1:**
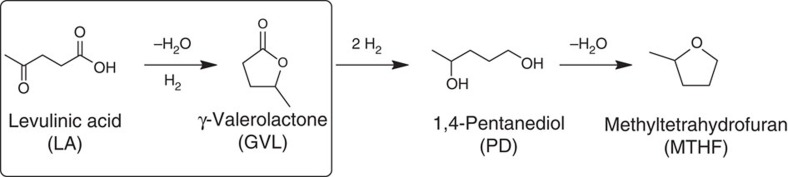
Catalytic conversion of levulinic acid. Reaction scheme for the catalytic conversion of the platform molecule levulinic acid (LA), which can be easily produced from the carbohydrate fraction in lignocellulosic biomass. Consecutive hydrogenation and dehydration steps can transform LA into the useful chemicals γ-valerolactone (GVL), 1,4-pentanediol (PD) and methyltetrahydrofuran (MTHF).

**Figure 2:**
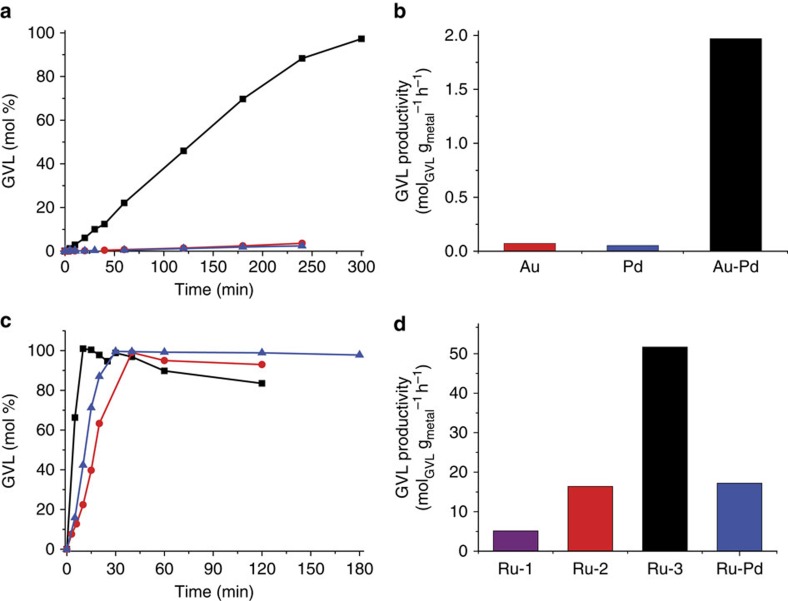
Catalytic performance of bimetallic and monometallic catalysts. (**a**) Production of GVL as a function of time during the hydrogenation of LA using monometallic 1% Au/TiO_2_ (*M*_Im_) (red circles) and 1% Pd/TiO_2_ (*M*_Im_) (blue triangles) and bimetallic 1% Au-Pd/TiO_2_ (*M*_Im_) (black squares) catalysts. (**b**) Comparison of GVL productivities of TiO_2_ supported Au, Pd and Au-Pd catalysts after 4 h of reaction time. (**c**) Production of GVL as a function of time during the hydrogenation of LA using monometallic 1% Ru/TiO_2_ (*M*_Im_) (red circles, Ru-2), 1% Ru/TiO_2_ (*M*_Im_, 0 M HCl) (black squares, Ru-3) and bimetallic 1% Ru-Pd/TiO_2_ (*M*_Im_) (blue triangles, Ru-Pd) catalysts. (**d**) Comparison of GVL productivities of supported 1% Ru/TiO_2_ (*W*_Im_) (Ru-1), 1% Ru/TiO_2_ (*M*_Im_) (Ru-2), 1% Ru/TiO_2_ (*M*_Im_, 0 M HCl) (Ru-3) and 1% Ru-Pd/TiO_2_ (*M*_Im_) (Ru-Pd) after 4 h of reaction time. Reaction conditions: *T*=473 K; *p*_H___2__=40 bar; 10 wt% LA in dioxane; LA to Ru weight ratio=1,000.

Initially, we tested 1% Au-Pd/TiO_2_ (*M*_Im_) under batch conditions with a 10 wt% solution of LA in dioxane at 473 K and 40 bar H_2_. The corresponding monometallic 1% Au/TiO_2_ (*M*_Im_) and 1% Pd/TiO_2_ (*M*_Im_) catalysts were also tested under identical conditions for comparison; all time-on-line yields of GVL (in mol%) are given in Fig. 2a. Complete LA consumption and product formation profiles are presented in Supplementary Figs 1–3. The monometallic Au and Pd catalysts were almost inactive with molar conversions reaching only 3.6 and 2.5% after 4 h, respectively. Consequently, GVL productivities, as shown in Fig. 2b, are very low at 0.073 mol_GVL _g_metal_^−1 ^h^−1^ (Au, TOF 0.004 s^−1^) and 0.054 mol_GVL _g_metal_^−1^ h^−1^ (Pd, TOF 0.005 s^−1^). In strong contrast, the bimetallic Au-Pd catalyst displayed a substantially higher catalytic activity under identical conditions, giving 90% conversion of LA at 97.5% GVL selectivity; LA conversion is quantitative after 5 h, with 97.3% selectivity to GVL. GVL productivity thus improved ~27-fold to 1.97 mol_GVL _g_metal_^−1 ^h^−1^ (TOF 0.1 s^−1^). Small amounts of PD (0.4%) and MTHF (0.3%) are formed as by-products by consecutive hydrodeoxygenation reactions (Fig. 1). Time-on-line data at different LA concentrations and hydrogen pressures are presented for 1% Au-Pd/TiO_2_ (M_Im_) in Supplementary Fig. 4.

Encouraged by this promising result and taking into account that Ru-based catalysts are typically most active in LA hydrogenation, we prepared a bimetallic 1% Ru-Pd/TiO_2_ (*M*_Im_) catalyst using the *M*_Im_ synthesis method and tested it under our standard reaction conditions. The results, including a comparison with various monometallic 1% Ru/TiO_2_ catalysts, are summarized in Fig. 2c,d, as well as in Supplementary Figs 5–8. With the bimetallic 1% Ru-Pd/TiO_2_ (*M*_Im_) catalyst after 30 min, quantitative conversion of LA (>99%) was observed with a very high GVL selectivity of 99.6%, corresponding to a productivity of 17.2 mol_GVL _g_metal_^−1 ^h^−1^ (TOF 0.6 s^−1^). The corresponding monometallic 1% Ru/TiO_2_ (*M*_Im_) catalyst gave quantitative LA conversion after 40 min with 99.0% selectivity to GVL (productivity of 16.4 mol_GVL _g_metal_^−1 ^h^−1^, TOF 0.5 s^−1^). Notably, GVL selectivity actually decreased to 93.0% with 1% Ru/TiO_2_ (M_Im_) at longer reaction times of up to 2 h, with PD (1.2%) and MTHF (0.7%) being formed as by-products (Fig. 1). In contrast, with the Ru-Pd catalyst, molar selectivity of GVL remained stable at~99% even after 2 h of reaction. Compared with the marked improvement in catalyst activity seen on alloying Au with Pd, the improvement in GVL productivity is more limited for Ru-Pd, but alloying did help in achieving and maintaining a very good GVL selectivity and, as importantly, improved the stability of the catalyst.

**Figure 3:**
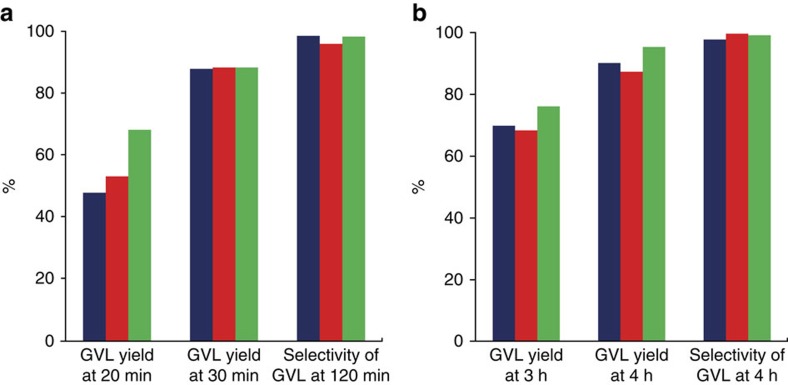
Recyclability tests of the bimetallic catalysts. Recyclability tests were performed at different LA conversion levels and GVL selectivities. No significant catalyst deactivation was detected after three consecutive catalytic runs. (**a**) 1% Ru-Pd/TiO_2_ (*M*_Im_). (**b**) 1% Au-Pd/TiO_2_ (*M*_Im_). The blue, red and green bars represent yields and selectivities obtained in the first, second and third catalytic runs, respectively.

The stabilities of the bimetallic 1% Ru-Pd/TiO_2_ (*M*_Im_) and 1% Au-Pd/TiO_2_ (*M*_Im_) catalysts were examined by performing three consecutive catalytic runs and the recyclability results of the catalysts are shown in Fig. 3. Catalytic activity and selectivity were also compared at different LA conversion levels. All results show that GVL selectivities and yields are sustained on multiple re-use. The limited leaching of the metal phase (Supplementary Table 2) and minimal loss of surface area (Supplementary Table 3) further illustrate that both bimetallic catalysts did not show significant deactivation during the three consecutive catalytic runs.

Although the addition of HCl to the precursor mixture is essential for generating alloys of uniform composition, it is not necessarily required for monometallic catalyst synthesis. We therefore also synthesized a 1% Ru/TiO_2_ (*M*_Im_, 0 M HCl) catalyst without using excess HCl. This catalyst, labelled as Ru-3 in Fig. 2d, showed exceptionally high activity. The GVL productivity of 51.7 mol_GVL _g_metal_^−1 ^h^−1^ (TOF 2.0 s^−1^) is much higher than the 16.4 mol_GVL _g_metal_^−1 ^h^−1^ obtained for 1% Ru/TiO_2_ (*M*_Im_) (TOF 0.5 s^−1^) prepared with an excess of 0.5 M HCl and labelled as Ru-2. The highly active 1% Ru/TiO_2_ (*M*_Im_, 0 M HCl) catalyst thus outperforms all catalysts tested by us^12^ and, as far as we are aware, others (Supplementary Table 1)^10^. As for 1% Ru/TiO_2_ (M_Im_), some sintering of the ruthenium nanoparticles was seen for the spent 1% Ru/TiO_2_ (*M*_Im_, 0 M HCl) catalyst, however. For comparison, we prepared a 1% Ru/TiO_2_ catalyst by conventional wet-impregnation followed by calcination and reduction, as previously reported^12^. The GVL productivity of this catalyst, labelled as Ru-1 and tested under identical reaction conditions, amounted to 5.14 mol_GV _ g_metal_^−1^ h^−1^ (TOF 0.2 s^−1^), a value more similar to the productivities of those supported Ru catalysts listed in a recent review article^10^. These results nonetheless demonstrate the importance of system-specific synthesis strategies to achieve exceptionally high catalytic activity.

### Metal particle size and alloy formation

**Figure 4:**
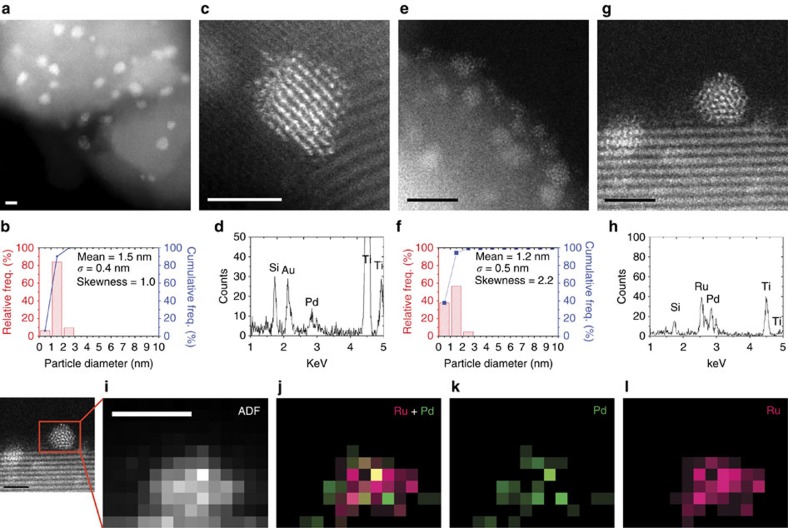
STEM analysis of the bimetallic catalysts. (**a**) HAADF image of 1% Au-Pd/TiO_2_ (*M*_Im_) showing typical particle sizes. (**b**) Corresponding particle size distribution derived from measurements of over 500 particles found on 1% Au-Pd/TiO_2_ (*M*_Im_). (**c**) HAADF image of an individual metal nanoparticle on 1% Au-Pd/TiO_2_ (*M*_Im_). (**d**) Its corresponding XEDS spectrum confirming it is a Au-Pd alloy. (**e**) HAADF image of 1% Ru-Pd/TiO_2_ (*M*_Im_) showing typical particle sizes. (**f**) Corresponding particle size distribution derived from measurements of over 500 particles on 1% Ru-Pd/TiO_2_ (*M*_Im_). (**g**) HAADF image of an individual metal nanoparticle on 1% Ru-Pd/TiO_2_ (*M*_Im_). (**h**) Its corresponding XEDS spectrum confirming it is a Ru-Pd alloy. (**i**) Zoom-in ADF image of an individual metal nanoparticle on 1% Ru-Pd/TiO_2_ (*M*_Im_). (**j**) Zoom-in elemental map of Ru (pink) and Pd (green). (**k**) Zoom-in Pd (green) elemental map. (**l**) Zoom-in Ru (pink) elemental map. The scale bar is 2 nm in all figures.

The composition and nanostructure of both bimetallic catalysts were studied by aberration-corrected STEM (AC-STEM). The mean size of the supported metal nanoparticles of the 1% Au-Pd/TiO_2_ (M_Im_) sample was 1.5 nm (Fig. 4a,b). X-ray Energy Dispersive Spectroscopy (XEDS) analysis confirmed these nanoparticles to be Au-Pd alloys (Fig. 4c,d). Small sub-nm clusters were also apparent in this material (Supplementary Fig. 9a), as were a limited amount of larger Au particles of about 0.1–0.5 μm in size (Supplementary Fig. 10). The mean size of the supported metal particles on 1% Ru-Pd/TiO_2_ (*M*_Im_) was slightly smaller at 1.2 nm (Fig. 4e,f), with XEDS analysis also confirming them to be Ru-Pd alloys (Fig. 4g–l). Many sub-nm clusters were again apparent (Supplementary Fig. 9b), but SEM analysis did not show any μm-scale particles for this sample (Supplementary Fig. 11).

**Figure 5:**
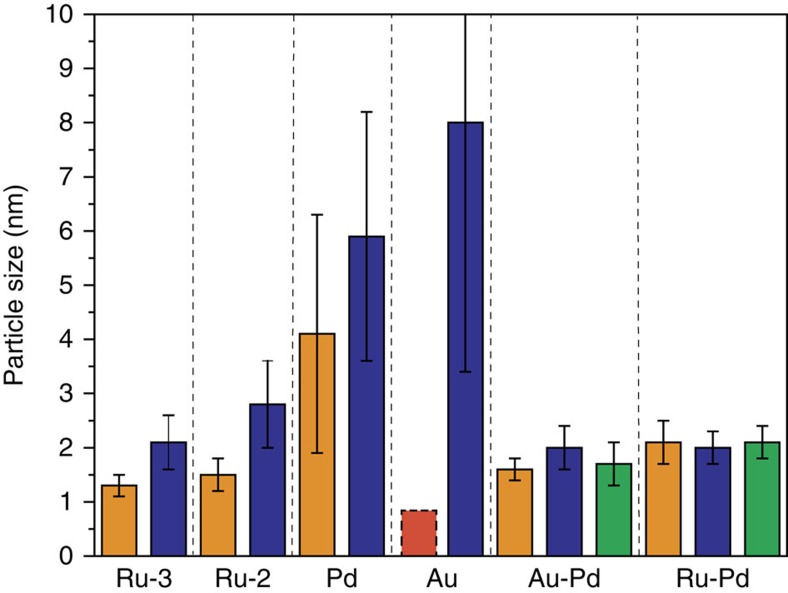
Mean metal particle sizes for the fresh and spent catalysts. Orange bar=fresh catalyst; Blue bar=spent catalyst after 1 run; Green bar=spent catalyst after three consecutive runs. Ru-2 and Ru-3 corresponds to 1% Ru/TiO_2_ (*M*_Im_) and 1% Ru/TiO_2_ (*M*_Im_, 0 M HCl), respectively. The red, dashed bar shown for the fresh 1% Au/TiO_2_ (*M*_Im_) catalyst indicates that sub-nanometre Au particles, that is, of a size below the TEM detection limit, are present, together with larger submicron-sized ones. All catalysts were prepared by the *M*_Im_ synthesis method. The error bars represent the standard deviation based on the histogram statistics for at least 200 counts of particle diameters.

Any changes in the mean metal particle diameters of the fresh and spent catalysts were examined by TEM (Fig. 5). All spent monometallic catalysts show an increase in the metal particle size and broadening of the distribution after the first catalytic run. In contrast, both bimetallic materials showed no or only a marginal increase in particle size, even after three catalytic runs, clearly illustrating the advantageous effect alloying has on stability.

To probe how representative the STEM results are for the entire catalyst samples, the materials were also characterized by extended X-ray absorption fine structure (EXAFS) measurements at the Au L_3_, Pd K and Ru K edges. The obtained results are summarized in Table 1, while the EXAFS spectra and associated Fourier Transform (FT) data can be found in Supplementary Figs 12,13. The EXAFS data indeed confirm the presence of bimetallic species and the bimetallic coordination numbers are given in Table 1. For 1% Au-Pd/TiO_2_ (M_Im_), the presence of bimetallic species is immediately evident from the FT data at both edges. Two intense peaks in the FTs are observed as a consequence of a ‘π phase flip’ in the backscattering amplitude from 6 Å^−1^ for Au, resulting in a splitting of the major contribution in the FT into a high and low *r* component^22^,^23^. This occurs when two elements are present in equivalent amounts; often the splitting and intensity of the low *r* contribution become more intense with an increasing number of bimetallic bonds. The mismatch in the total coordination number from an analysis of the Au L_3_-edge (7.3) and Pd (3.0) K-edge data is testament to a large number of Au-Au contributions due to the additional presence of larger (~μm sized) Au-rich particles, as also seen by STEM–XEDS. From a Pd K-edge perspective, the smaller overall coordination number suggests that much of the Pd is associated with Au. For the 1% Ru-Pd/TiO_2_ (*M*_Im_) sample, the similarity in X-ray scattering efficiency precludes distinguishing between the bimetal components. However, instead, a first shell analysis assuming one metal-metal (M-M) scatterer type can be performed on the basis that STEM–XEDS confirms the bimetallic nature of the metal particles for this sample. The closeness in the coordination numbers of both elements from an analysis of both edges supports the notion that the Pd and Ru exist within intimately mixed bimetallic particles. The large number of M-O bond distances determined and the low M-M coordination numbers seen for both edges are also consistent with the STEM analysis in that the particles are very small, smaller than that seen for the Au-Pd sample. In addition, the shorter distances of the Ru-O and Ru-Ru bonds with 1% Ru/TiO_2_ (M_Im_, 0 M HCl) as compared with 1% Ru-Pd/TiO_2_ (*M*_Im_) (Table 1) indicate that even smaller particles are present in the 1% Ru/TiO_2_ (*M*_Im_, 0 M HCl) catalyst, corroborating the TEM results.

**Figure 6:**
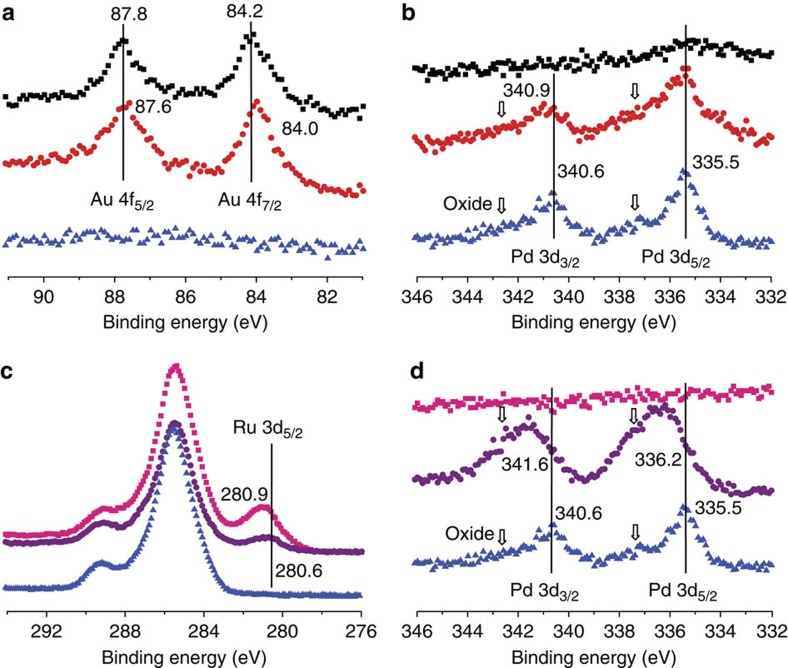
XPS spectra of selected monometallic and bimetallic *M*_Im_ catalysts. (**a**) Au 4f spectral region. (**b**) Pd 3d spectral region. The arrows indicate the presence of some Pd oxide in the sample. (**c**) Ru 3d spectral region. (**d**) Pd 3d spectral region. Catalyst sample codes are as follows: (pink squares) 1% Ru/TiO_2_ (*M*_Im_, 0 M HCl); (purple circles) 1% Ru-Pd/TiO_2_ (*M*_Im_); (blue triangles) 1%Pd/TiO_2_ (*M*_Im_); (black squares) 1% Au/TiO_2_ (M_Im_) and (red circles) 1% AuPd/TiO_2_ (*M*_Im_).

All fresh monometallic and bimetallic catalysts were also characterized by XPS. Figure 6 shows the Au 4 f, Ru 3d and Pd 3d XPS spectra of a selection of mono- and bimetallic catalysts, while the Ti 2p and O 1s XPS spectra can be found in Supplementary Fig. 14. Supplementary Table 4 summarizes the measured XPS binding energies (BE) for the different *M*_Im_ catalysts. For 1% Au/TiO_2_ (*M*_Im_), the Au 4f_7/2_ signal is observed at 84.2 eV (Fig. 6a), which is close to the binding energies previously reported for Au/TiO_2_ (refs 24, 25, 26) and Au/Fe_2_O_3_ samples^27^. The same signal showed a small negative shift of −0.2 eV, from 84.2 to 84.0 eV for 1% Au-Pd/TiO_2_ (M_Im_). Similar small negative shifts of −0.25 eV to −0.56 eV in Au4f_7/2_ BE’s have previously been observed for Au-Pd surfaces and nanoparticles^28^,^29^,^30^,^31^. This has been attributed to negative charge build-up on the Au atoms on Au-Pd alloy formation as a result of Au (2.4) being more electronegative than Pd (2.2)^30^. The positive shift of +0.3 eV from 340.6 to 340.9 eV seen for the Pd 3d_3/2_ peak (Fig. 6b) is in line with this and suggests deposition of some positive charge onto the Pd atoms. Alternatively, the positive shift could also result from partial oxidation of Pd. It should be kept in mind that the EXAFS data show that Pd is predominantly present in Au-Pd particles, but metallic Pd and Pd-O species were also detected. The latter were also seen for Au-Pd/SiO_2_ catalysts^32^. Indeed, both the 1% Au-Pd/TiO_2_ (M_Im_) and 1% Pd/TiO_2_ (M_Im_) samples showed weak peaks at around 342.6 (ref. 27) and 337.2 eV (ref. 33), confirming the presence of Pd oxide on the surface of both samples. The Ti 2p and O 1s XPS peaks were observed at the same BE for the Au, Pd and Au-Pd catalysts, suggesting a similar level of interaction between the metal and TiO_2_ support in these three catalyst materials (Supplementary Fig. 14).

For the Ru-based catalyst samples, the overlap of the Ru 3d_3/2_ and Ru^n+^ 3d_5/2_ XPS signals with the C 1s sample holder XPS signal (Fig. 6c) allows the metallic state of Ru to be studied only via the Ru 3d_5/2_ XPS signal. Elmasides *et al*.^34^ previously reported this XPS peak to be at 280.1 eV for Ru^0^ supported on TiO_2_. For 1% Ru/TiO_2_ (M_Im_, 0 M HCl), a positive shift of +0.8 eV of the Ru 3d_5/2_ signal to 280.9 eV was observed, together with negative shifts of both the Ti 2p and O 1s XPS peaks; in addition, shoulder peaks appeared on the Ti 2p and O 1s XPS signals, which point at electron transfer from Ru to TiO_2_ with concomitant reduction of some of the Ti^4+^ into Ti^3+^. For the Ru-Pd sample, the Ru 3d_5/2_ peak is instead observed at 280.6 eV. This, together with the smaller changes in the Ti 2p and O 1s XPS spectra (Supplementary Fig. 14), indicates that the interaction between Ru and TiO_2_ is weaker for the Ru-Pd sample than in 1% Ru/TiO_2_ (*M*_Im_, 0 M HCl). The Pd 3d_5/2_ (+0.9 eV) and Pd 3d_3/2_ (+1.0 eV) peaks furthermore show a positive shift and larger Pd oxide contribution compared with 1% Pd/TiO_2_ (*M*_Im_), indicative of highly dispersed and positively charged Pd species on the surface of the 1% Ru-Pd/TiO_2_ (*M*_Im_) catalyst. EXAFS and XPS thus both show a more pronounced Pd-O character in 1% Ru-Pd/TiO_2_ (M_Im_). All this thus suggests that at the metal surface Ru is less positively charged as a result of a weaker interaction of Ru with the support, while Pd is highly positively charged with some Pd-O character at the surface. Note that no residual Cl was detected by XPS in any of the monometallic and bimetallic *M*_Im_ catalysts under investigation, except for a trace amount in the 1% Au/TiO_2_ (*M*_Im_) sample.

### CO FT-IR spectroscopy

**Figure 7:**
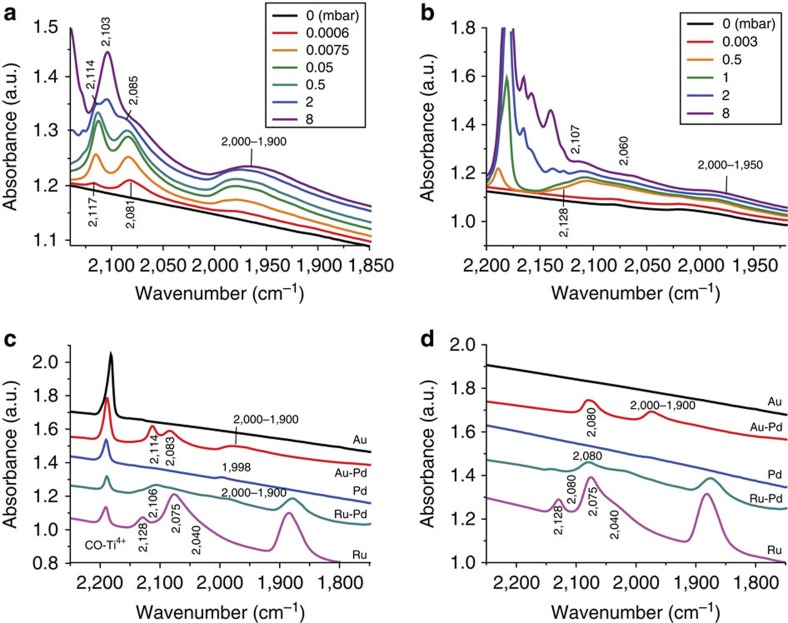
FT-IR spectra of CO adsorption and desorption on monometallic and bimetallic catalysts. (**a**) 1% Au-Pd/TiO_2_ (*M*_Im_) catalyst measured at 87 K at different CO pressures. (**b**) 1% Ru-Pd/TiO_2_ (*M*_Im_) catalyst measured at 87 K and different CO pressures. (**c**) FT-IR spectra measured at 87 K and a partial CO pressure of 0.5 mbar of 1% Au/TiO_2_(*M*_Im_), 1% Pd/TiO_2_(*M*_Im_), 1% Ru/TiO_2_ (*M*_Im_, 0 M HCl), 1% Au-Pd/TiO_2_ (*M*_Im_) and 1% Ru-Pd/TiO_2_ (*M*_Im_) catalysts (**d**) at 87 K and *p*_CO_=0.5 mbar; (**d**) FT-IR spectra measured at 473 K and a *p*_CO_ of~10^−6 ^mbar for the same set of catalysts as under **c**.

All mono- and bimetallic catalysts were also characterized by FT-IR spectroscopy after CO adsorption, see also Supplementary Figs 15–19. Figure 7a shows the changes in the FT-IR spectra of adsorbed CO over 1% Au-Pd/TiO_2_ (*M*_Im_) at 87 K on stepwise increase in CO pressure. The three spectral features at 2,117, 2,081 and 2,000–1,900 cm^−1^, which are already detected at low CO pressures, can be assigned to linear Au-CO, linear Pd-CO and bridging Pd-CO species, respectively^35^,^36^,^37^,^38^,^39^,^40^. Notably, a feature similar to the one at 2,081 cm^−1^ has been ascribed to CO linearly adsorbed on isolated Pd atoms in Au-Pd alloy films^40^. Furthermore, a red shift similar to the shift of the Au-CO(L) feature at 2,117 to 2,114 cm^−1^ on increasing the CO pressure has also been seen in Au-Pd model catalyst systems^39^,^40^,^41^,^42^. The simultaneous blue-shift of the Pd-CO(L) signal indicates strong electronic interaction between these Au-CO(L) and Pd-CO(L) species and again point at the direct adjacency of Au and Pd atoms. The new, weakly adsorbed CO species that is observed at 2,103 cm^−1^ at higher *p*_CO_ can be assigned to CO adsorption on isolated monometallic Pd species, which were also detected by STEM^35^,^40^,^43^.

Taken together, the partial negative charge on Au as seen in XPS, the confirmation of an Au-Pd nano-alloy by STEM and EXAFS, the evidence obtained by FT-IR for isolated Pd species and the close proximity of Au and Pd, all suggest efficient mixing and electron transfer from Pd to Au in the Au-Pd catalyst. The fact that CO adsorption on Pd is much stronger on Au-Pd alloy formation (Fig. 7c, d), also seen before with a model Au-Pd catalyst^40^, provides further evidence of the strong electronic modification of Pd upon nano-alloy formation with Au. This electronic modification of Pd is considered the cause of the marked increase in LA hydrogenation activity^13^,^44^. Changes in surface reactivity of bimetallic nano-alloys, such as the one observed for the Au-Pd system, is thought to be the consequence of a combination of electronic and geometric effects. Indeed, to disentangle the relative contributions to alloying is a difficult task. For Au-Pd systems, DFT studies have suggested that on incorporation of larger Au atoms in the Pd lattice (with has a smaller lattice constant than Au), a lattice mismatch results in tensile strain in the structure of these Au-Pd nano-alloys. This strain generally induces changes in the ability of this metal (active) site to form bonds to adsorbed species or, in other words, changes the reactivity of the metal (active) sites^45^. This change in activity can be thought to be the result of a change in width and centre of the d-band centres (for both Au and Pd) on alloying^46^. In this case, a narrowing and upward shift of the d-band of Pd due to the tensile strain would then result in enhanced chemical reactivity of these surface atoms. Further computational studies are, however, needed to understand what is the exact cause of the synergistic effect reported here.

Differentiation of the adsorbed species on 1% Ru-Pd/TiO_2_ (*M*_Im_) proved more difficult, as a result of overlapping of bands of CO adsorbed on Ru and Pd. Furthermore, little electron transfer between Ru and Pd is expected given that the electronegativity of the two is the same. In line with the XPS and EXAFS data, the FT-IR spectra of Fig. 7b do, nonetheless, support that Ru and oxidized Pd are present in a Ru-Pd nano-alloy catalyst materials.

Notably, less CO is adsorbed on the Ru-Pd catalyst than on 1% Ru/TiO_2_ (*M*_Im_, 0 M HCl) at 473 K and under high vacuum, as can be seen from the attenuated intensity of the Ru^δ+^-CO(L) vibration at 2,075 cm^−1^ in the FT-IR spectra of Supplementary Figs 18,19^24^,^25^,^26^. This indicates a weakened interaction between Ru and TiO_2_ in the bimetallic sample, as is also evidenced by the longer Ru-O bond lengths found by EXAFS and the more limited partial Ti reduction seen by XPS (Fig. 6, Supplementary Fig. 14 and Supplementary Table 4). These observations provide further insight into the improved performance of the 1% Ru-Pd/TiO_2_(*M*_Im_) catalyst. First, the lack of sintering of Ru is suggested to be the result of a stabilization effect^29^,^44^, in which the presence of Pd species on the surface dilutes and isolates the active Ru sites; second, the catalyst can maintain its excellent GVL selectivity, as the weakened interaction between support and metal effectively ‘switches-off’ any consecutive hydrogenation reactions.

**Figure 8:**
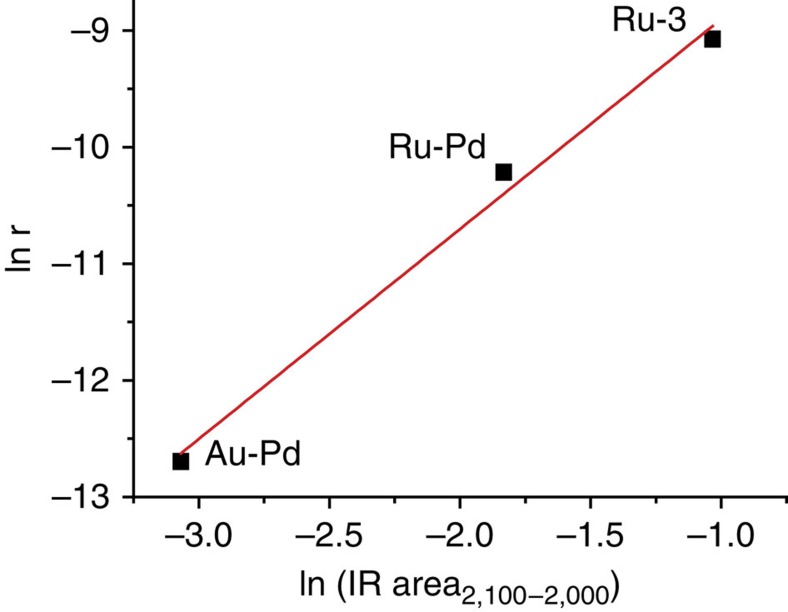
Relationship between reaction rate and adsorbed linear CO species for different catalysts. Plot of the natural logarithm of reaction rate versus amount of adsorbed linear CO species for different catalysts at 473 K, the reaction temperature for the LA to GVL conversion. The initial reaction rate is expressed in mol s^−1^ and the amount of linearly adsorbed CO was determined by integration of the spectra between 2,100 cm^−1^ and 2,000 cm^−1^, normalized by pellet weight.

Finally, fitting the CO adsorption data with a Temkin-Pyzhev adsorption isotherm (Θ=a ln (b × *p*_CO_) where Θ is the coverage of CO, and a and b are constants), as illustrated in Supplementary Fig. 20, revealed a semi-quantitative correlation of CO adsorption with catalytic activity. Indeed, Fig. 8 shows that the initial rate of LA to GVL transformation for three different catalysts was found to correlate well with the amount of linearly CO adsorbed at the actual reaction temperature. Similar CO extinction coefficients were assumed for the linearly adsorbed CO species detected in the 2,100–2,000 cm^−1^. Interestingly, Fan *et al*.^47^ reported that small amounts of CO (~1,000 p.p.m.) already had a significant, negative effect on LA hydrogenation over supported platinum-group metals, suggesting that the active sites for both CO adsorption and LA to GVL hydrogenation are indeed related. Consequently, we believe that CO adsorption, as measured by FT-IR, can be used to semi-quantitatively predict catalyst activity for this reaction. Further studies, spanning a broader range of catalysts, are required to further substantiate this correlation.

## Discussion

In summary, TiO_2_-supported bimetallic Au-Pd and Ru-Pd nano-alloy catalysts were synthesized by a modified impregnation (*M*_Im_) method and shown to be highly active in the hydrogenation of levulinic acid to γ−valerolactone. Alloying was found to have two distinct effects. First, an electronic effect is seen for 1% Au-Pd/TiO_2_, with catalytic activity resulting from the modification of the electronic properties of Pd upon alloying with Au. The combination of two relatively inactive metals thus led to a remarkable increase in catalytic activity. Second, a stabilization effect is seen for 1% Ru-Pd/TiO_2_, with Pd species diluting and isolating the active Ru sites, resulting in excellent selectivity to GVL by switching-off consecutive hydrogenation reactions. Both the Ru-Pd and Au-Pd nano-alloy catalysts furthermore showed excellent stability even after three consecutive catalytic runs. Finally, a variation in the synthesis method of the benchmark monometallic 1% Ru/TiO_2_ catalyst led to the discovery of an exceptionally active catalyst (51.7 mol_GVL _g^−1^_metal_ h^−1^ and TOF=2.0 s^−1^). In contrast to the bimetallic Ru-Pd catalyst, limited sintering was observed though for both monometallic Ru catalysts, that is, the exceptionally active and the MIm prepared one. These findings open up new possibilities for the application of bimetallic catalysts, prepared using carefully designed synthesis strategies, in bio-based fuel and chemicals production. Future studies should now be directed at an assessment of longer-term stability, that is, under continuous-flow conditions, as well as at a more detailed understanding of the two distinct effects of metal alloying, for example, by computational means.

## Methods

### Catalyst preparation

All the supported monometallic and bimetallic catalysts were prepared from their corresponding metal chloride precursors via a modified wet-impregnation method that involves using an excess of chloride anions (*M*_Im_)^20^ All catalysts prepared by this modified impregnation method are represented as *M*_Im_ catalysts. The *M*_Im_ method entails the use of the following precursor solutions: HAuCl_4_.xH_2_O (>99.9%, Sigma Aldrich) was dissolved in deionized water to form a solution with a Au concentration of 9.34 mg ml^−1^. RuCl_3_ (99.9%, Acros Chemicals) was dissolved in deionized water to form an aqueous solution with a Ru concentration of 5.28 mg ml^−1^. The PdCl_2_ salt (<99%, Sigma Aldrich) was dissolved in 0.5 M HCl under vigorous stirring and gentle warming to obtain a solution with a resultant Pd concentration of 3.02 mg ml^−1^. This solution was slowly cooled and used as the Pd precursor.

In a typical catalyst synthesis, the requisite amount of precursor solution(s) was charged into a clean 50 ml round bottomed flask fitted with a magnetic stirrer, after which the requisite amount of concentrated HCl (37.5%) was added and the volume of the precursor/HCl solution finally adjusted to 25 ml to obtain a final HCl concentration of 0.5 M in deionized water. The round bottom flask was submerged in a temperature-controlled oil bath and the mixture was then agitated vigorously at 298 K using a hot plate stirrer. To the stirred precursor solution, the requisite amount of the support (P-25 TiO_2_, Evonik-Degussa) was added very slowly with constant stirring at 298 K over nearly 30 min. After complete support addition, the slurry was stirred vigorously and then the temperature was raised to 358 K. The slurry was stirred at this temperature overnight until all water had evaporated. The solid powder obtained, denoted as the ‘dried sample’, was ground thoroughly and then reduced in a furnace at 723 K (~2 K min^−1^ ramp rate) under a flow of 5% H_2_/He for 4 h. All the monometallic and bimetallic catalysts were prepared with a 1 wt% (total) metal content on a 1 g scale and in the case of the bimetallic *M*_Im_ catalysts, the two metals were added in an equimolar metal ratio.

The 1% Ru/TiO_2_ (*M*_Im_, 0 M HCl) catalyst, without the addition of HCl, was prepared with similar methodology. The requisite amount of the precursor RuCl_3_ solution was charged into a clean 50 ml round-bottomed flask fitted with a magnetic stirrer and then the solution was made up to 25 ml with deionized water, and no HCl was added. Subsequent steps were identical to those mentioned in the previous *M*_Im_ method.

### Catalyst testing

All the catalytic hydrogenation reactions of LA to GVL were performed in dioxane using a high-pressure autoclave reactor fitted with an overhead stirrer. The reactions were run in a 100 ml Parr batch autoclave at a temperature of 473 K for 10 h using a hydrogen pressure of 40 bar and a stirring speed of 1,600 r.p.m. Reactions were performed with 10 wt% levulinic acid (6.0 g, 51.7 mmol) in dioxane (54 g) with 1 wt% of catalyst (0.6 g). One millilitre of solution was sampled at various intervals during the reaction. Before the reaction, the batch autoclave reactor was loaded with catalyst, substrate and solvent, purged three times with argon after which the reaction mixture was heated to reaction temperature and charged with H_2_ to 40 bar. This was taken as the starting point of the reaction. After the reaction was cooled to room temperature, the H_2_ pressure was released and 2 wt% anisole was added as an internal standard. The catalyst was separated by centrifugation, filtration and finally washed with acetone.

The reaction products were analysed using a Shimadzu GC-2010 A gas chromatograph equipped with a CP-WAX 57-CB column (25 m × 0.2 mm × 0.2 μm) and FID detector. Products were identified with a GC−MS from Shimadzu with a CP-WAX 57CB column (30 m × 0.2 mm × 0.2 μm). The gas phase reaction products were analysed by an on-line dual channel Varian CP4900 micro-GC equipped with a COX column and TCD detector, for analysis of H_2_, CO_2_, CO and CH_4_. The turnover frequency (TOF) values were calculated as follows: TOF=initial rate/(metal amount × dispersion of metal × time). The influence of external mass transfer limitations on catalysis was studied as a function of stirrer speed with the benchmark Ru/C catalyst. Such limitations could be overcome if stirring speeds were set higher than 900 r.p.m. and all experiments reported in this article were subsequently run at a stirrer speed of 1,600 r.p.m.

### Catalyst characterization

Samples for examination by scanning transmission electron microscopy (STEM) were prepared by dispersing the dry catalyst powder onto a holey carbon film supported by a 300 mesh copper TEM grid. STEM high angle annular dark field (HAADF) images of the supported metal nanoparticles were obtained using an aberration corrected JEM ARM-200 F STEM operating at 200 kV. X-ray energy dispersive (XEDS) spectra were acquired from individual metal nanoparticles >1 nm in size by rastering the beam over the entire metal particle, while using a JEOL Centurio 0.9sr silicon drift detector. The sample powders were also dispersed onto an Al-stub and examined in SE and backscatter mode in a Hitachi 4300LV scanning electron microscope (SEM) equipped with an EDAX energy dispersive X-ray spectrometer to determine if there were any μm-scale metal particles present.

Transmission electron microscopy (TEM) measurements were conducted in bright field imaging mode using a Tecnai 20FEG transmission electron microscope operating at 200 kV. The mean Ru particle diameters were calculated by determining the size of more than 200 particles per sample using iTEM software (soft Imaging System GmbH). For non-symmetrical particle shapes, both the largest and shortest diameter was measured to obtain an average value.

Metal dispersion has been estimated by TEM/STEM: D=6 × (*ν*_m_/*a*_m_)/*d*, where *ν*_m_ is bulk metal atomic density of Ru (13.65 × 10^−3^ nm^3^), Pd (14.7 × 10^−3^ nm^3^) and Au (16.94 × 10^−3^ nm^3^), *a*_m_ is the surface area occupied by an atom on a polycrystalline surface of Ru (6.35 × 10^−2^ nm^2^), Pd (7.93 × 10^−2^ nm^2^) and Au (8.75 × 10^−2^ nm^2^) and *d* is the cluster size of metal determined by TEM and STEM.

X-ray absorption fine structure (XAFS) measurements were performed on station BM26A and BM23 at the ESRF^48^. The measurements were carried out in air on self-supporting wafers in transmission mode using a Si(111) monochromator at the Au L_3_-edge, Pd K-edge and Ru K-edge with the respective monometallic foils used as reference materials. All data were subjected to background correction using Athena (that is, IFFEFFIT software package) followed by either single or dual shell EXAFS fitting analyses performed using the DL-EXCURV program^49^,^50^.

X-ray photoelectron spectroscopy (XPS) measurements were carried out on a Thermo Scientific K-Alpha, equipped with a monochromatic small-spot X-ray source and a 180° double focusing hemispherical analyser with a 128-channel detector. Spectra were obtained using an aluminium anode (Al *K*_α_=1486.6 eV) operating at 72 W and a spot size of 400 μm. Survey scans were measured at a constant pass energy of 200 eV and more detailed region scans at a pass energy of 50 eV. The background pressure of the system was 2 × 10^−9^ mbar, and during measurement it was 3 × 10^−7^ mbar (of argon) because of the charge compensation dual beam source.

N_2_ physisorption isotherms were recorded to determine surface areas and pore volumes using a Micromeritics Tristar 3000 set-up operating at 77 K. Bimetallic catalysts were outgassed for 12 h at 473 K in a nitrogen flow before the physisorption measurements. BET surface areas were determined using 10 points between 0.06 and 0.25. Micropore volumes (cm^3 ^g^−1^) were determined by *t*-plot analysis for *t* between 3.5 and 5.0 Å to ensure inclusion of the minimum required pressure points.

FT-IR spectra were recorded on a Perkin-Elmer 2000 instrument with each spectrum consisting of 25 scans recorded at a resolution of 4 cm^−1^. Self-supported catalyst wafers (37±10 mg per 16 mm) were pressed at 3 kbar pressure for 10 s. The wafer was placed inside a synchrotron cell with a CaF_2_ window. The cell was evacuated to 10^−6 ^mbar, and the sample was subsequently dried at 573 K (3 K min^−1^) for 1 h. The cell was cooled down to 87 K with liquid nitrogen and connected to a gas chamber that permitted adjustment of the pressure of CO injected into the cell. CO adsorption was studied at 87 K at stepwise increasing pressures with fresh catalysts previously reduced in H_2_ at 723 K. CO desorption was also studied at 87 K during the evacuation process, and followed by temperature programmed desorption (3 K min^−1^) from 87 to 573 K for 30 min under high-vacuum conditions (~10^−6 ^mbar).

## Author contributions

W.L. performed the catalytic experiments, carried out the FT-IR experiments and analysed the XPS data under the guidance of P.C.A.B. and B.M.W. M.S. prepared all the MIm catalysts, while A.M.B., M.S. and W.L. performed the XAS measurements and A.M.B. analysed the EXAFS data; Q.H. performed the STEM and XEDS measurements under the guidance of C.J.K. All authors contributed to the discussion and manuscript preparation. P.C.A.B. and B.M.W. supervised the project.

## Additional information

**How to cite this article**: Luo, W. *et al*. High performing and stable supported nano-alloys for the catalytic hydrogenation of levulinic acid to γ-valerolactone. *Nat. Commun*. 6:6540 doi: 10.1038/ncomms7540 (2015).

## Supplementary Material

Supplementary Information Supplementary Figures 1-20, Supplementary Tables 1-4 and Supplementary References

## Figures and Tables

**Table 1:** EXAFS parameters.

Sample	Au-Au R (Å)	(N)	2σ^2^ (Å^2^)	Au-Pd R (Å)	(N)	2σ^2^ (Å^2^)	Pd-Pd R (Å)	(N)	2σ^2^ (Å^2^)	Pd-Au R (Å)	(N)	2σ^2^ (Å^2^)
1% AuPd/TiO_2_ (M_Im_)	2.80	4.8	0.017	2.77	2.5	0.014	2.75	1	0.014	2.78	2.0	0.016
1% Au/TiO_2_ (M_Im_)	2.84	10.0	0.017									
	**Ru-O**			**Ru-Ru (Pd)**			**Pd-O**			**Pd-Pd (Ru)**		
1% RuPd/TiO_2_ (*M*_Im_)	2.0	3.0	0.011	2.69	2	0.023	2.0	1.7	0.009	2.72	1.8	0.019
1% Pd/TiO_2_ (*M*_Im_)							2.0	2.0	0.010	2.73	4.0	0.019
1% Ru/TiO_2_ (*M*_Im_ 0 M HCl)	1.96	2	0.007	2.67	6	0.013						

EXAFS parameters for the monometallic and bimetallic catalysts determined from an analysis of the Au L_3_, Pd K and Ru K edges. *E*_f_~±15 eV; *R*-values for all data range from 28–38%; Afac values, 0.94 (Pd/Ru) and 0.98 Au. The Debye–Waller factors were initially determined from the 1% Au-Pd/TiO_2_ (*M*_Im_) sample and not refined for the remaining Au-Pd samples. For sample 1% AuPd/TiO_2_ (*M*_Im_) a Pd-O contribution is also present at 2.02 Å with *N*=1 and 2*σ*^2^**=**0.0013 Å^2^.
