# Supplementary material for: High performing and stable supported nano-alloys for the catalytic hydrogenation of levulinic acid to γ-valerolactone
Source: Nat Commun. 2015 Mar 17;6:6540. doi: 10.1038/ncomms7540 (PMC4382690; doi:10.1038/ncomms7540)
Supplement: Supplementary Information — Supplementary Figures 1-20, Supplementary Tables 1-4 and Supplementary References [file ncomms7540-s1.pdf]

Supplementary Figures

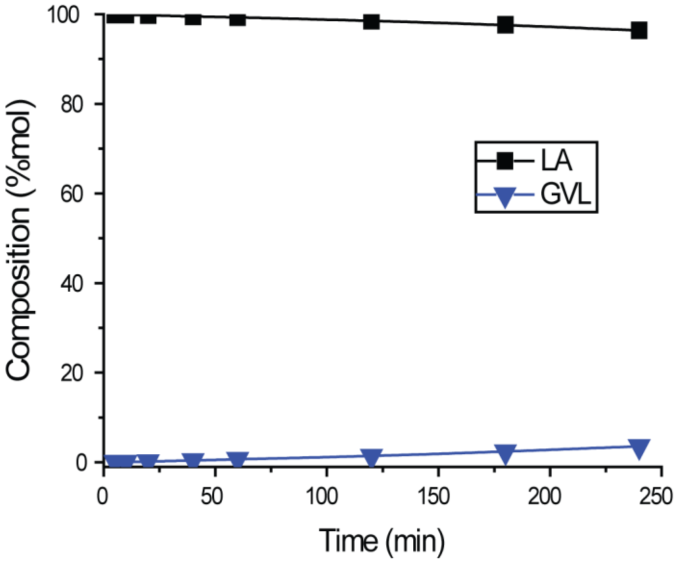

Supplementary Figure 1: Temporal evolution of GVL (green triangles) and LA (black squares) during the catalytic hydrogenation of LA over 1% Au/TiO_2 (M_{Im}). Reaction conditions: T: 473 K, P: 40 bar H_2, 10 wt% LA in dioxane, and a substrate/metal weight ratio of 1000.

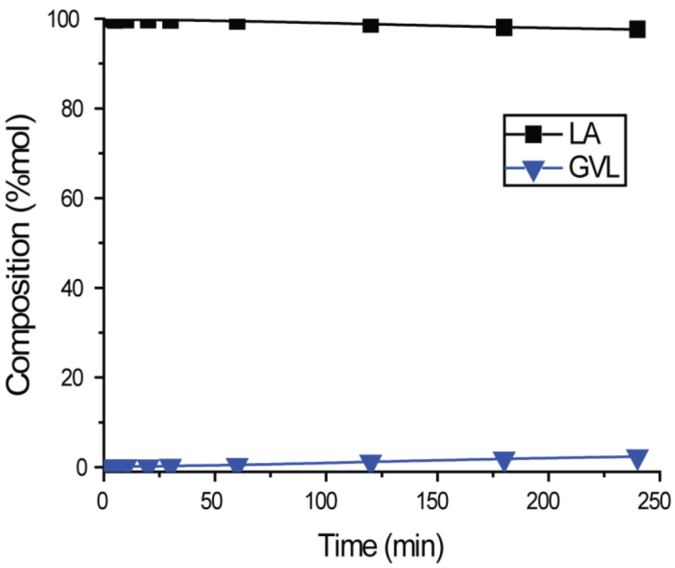

Supplementary Figure 2: **Temporal evolution of GVL (green triangles) and LA (black squares) during the catalytic hydrogenation of LA over 1% Pd/TiO_2 (M_{Im}).** Reaction conditions: T: 473 K, P: 40 bar H_2, 10 wt% LA in dioxane, and a substrate/metal weight ratio of 1000.

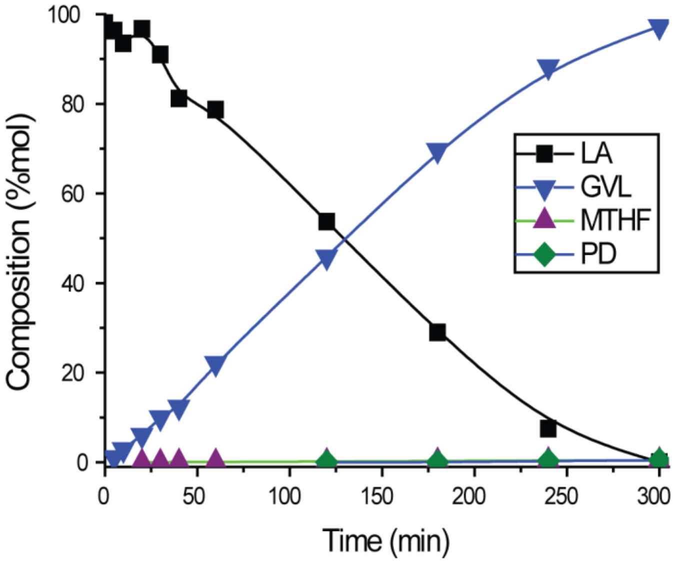

Supplementary Figure 3: **Temporal evolution of GVL (blue triangles), MTHF (purple triangles), PD (green diamonds) and LA (black squares) during the catalytic hydrogenation of LA over 1% Au-Pd/TiO_2 (M_{Im}).** Reaction conditions: T: 473 K, P: 40 bar H_2, 10 wt% LA in dioxane, and a substrate/metal weight ratio of 1000.

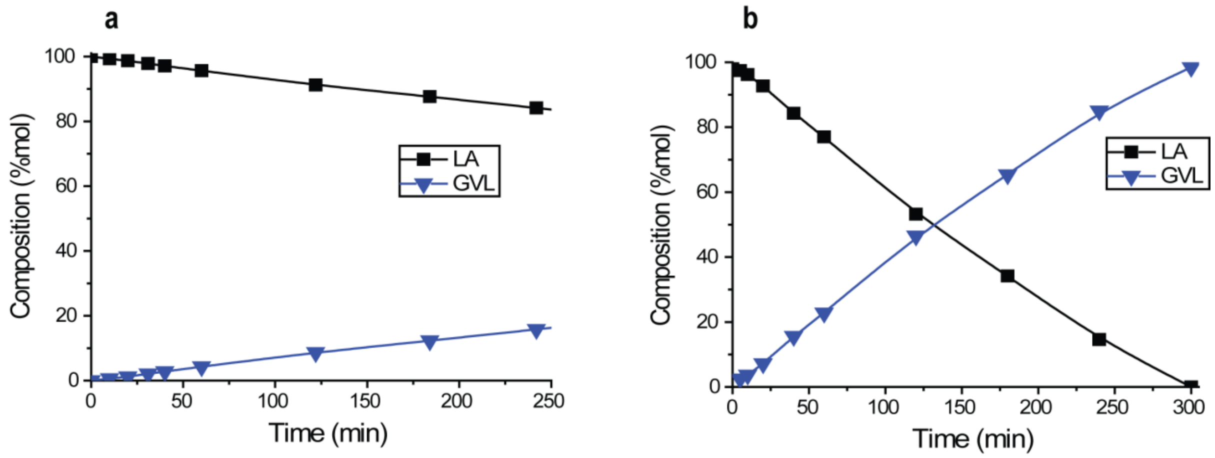

Supplementary Figure 4: Temporal evolution of GVL (blue triangles) and LA (black squares) during the catalytic hydrogenation of LA over bimetallic 1% Au-Pd/TiO_2 (M_{Im}). (**a**) at a higher levulinic acid loading of 20 wt% (reaction conditions: T: 473 K; P: 40 bar H_2; 20 wt% LA in dioxane, and a substrate/metal weight ratio of 2000). (**b**) at a higher hydrogen pressure of 60 bar (reaction conditions: T: 473 K, P: 60 bar H_2, 10 wt% LA in dioxane, and a substrate/metal weight ratio of 1000).

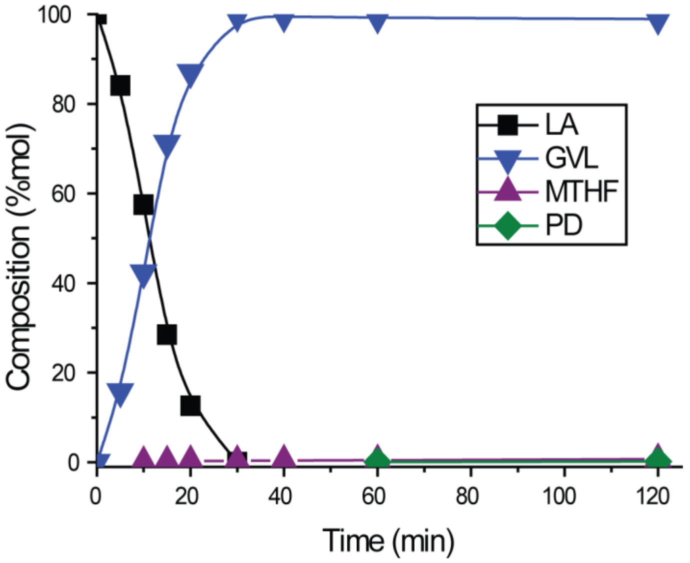

Supplementary Figure 5: **Temporal evolution of GVL (blue triangles), MTHF (*purple triangles*), PD (green diamonds) and LA (black squares) during the catalytic hydrogenation of LA over bimetallic 1% Ru-Pd/TiO_2 (M_{Im}).** Reaction conditions: T: 473 K, P: 40 bar H_2, 10 wt% LA in dioxane, and a substrate/metal weight ratio of 1000.

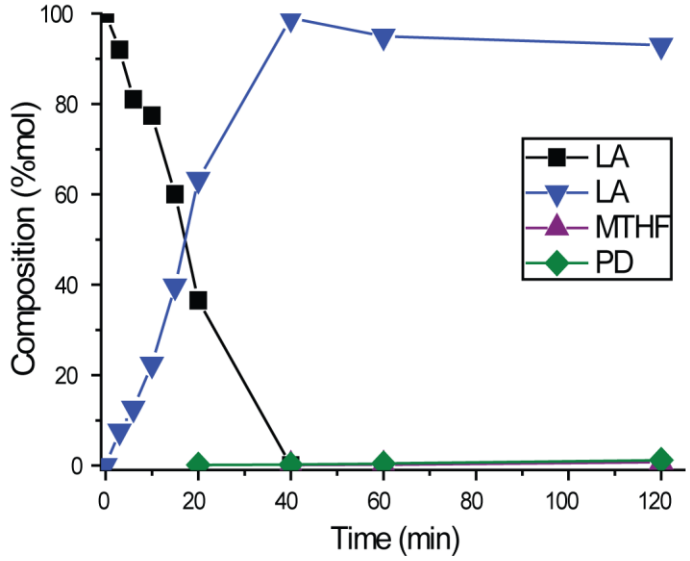

Supplementary Figure 6: **Temporal evolution of GVL (blue triangles), MTHF (*purple triangles*), PD (green diamonds) and LA (black squares) during the catalytic hydrogenation over monometallic 1% Ru/TiO_2 (M_{Im}).** Reaction conditions: T: 473 K, P: 40 bar H_2, 10 wt% LA in dioxane, and a substrate/metal weight ratio of 1000.

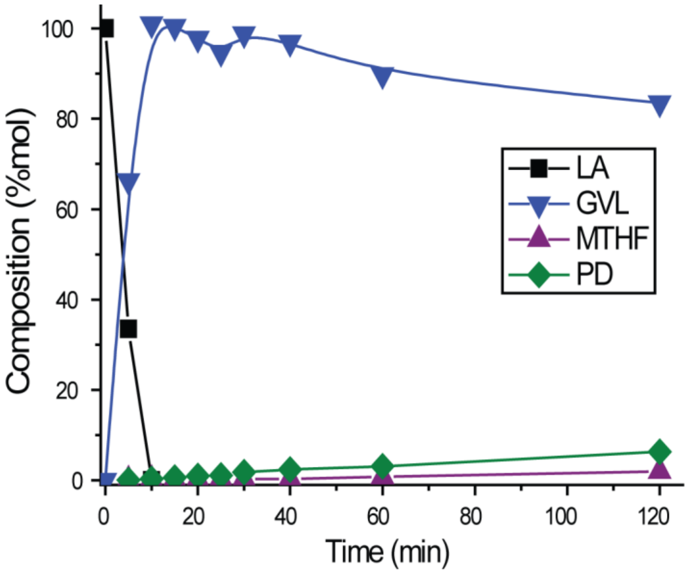

Supplementary Figure 7: Temporal evolution of GVL (blue triangles), MTHF (purple triangles), PD (green diamonds) and LA (black squares) during the catalytic hydrogenation of LA over monometallic 1% Ru/TiO_2 (M_{Im}, 0 M HCl). Reaction conditions: T: 473 K, P: 40 bar H_2, 10 wt% LA in dioxane, and a substrate/metal weight ratio of 1000.

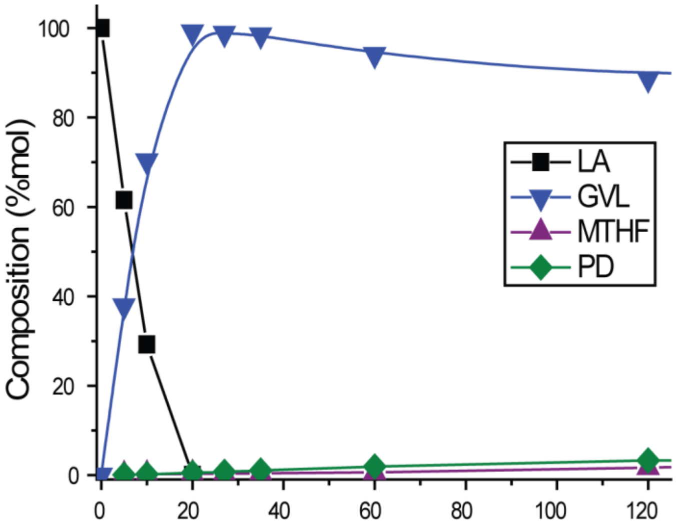

Supplementary Figure 8: Temporal evolution of GVL (blue triangles), MTHF (purple triangles), PD (green diamonds) and LA (black squares) during the catalytic hydrogenation of LA over monometallic 1% Ru/TiO_2 (M_{Im}, 0 M HCl) with the same the same Ru loading as in the run with the bimetallic 1% Ru-Pd/TiO_2 (M_{Im}) catalyst. Reaction conditions: T: 473 K, P: 40 bar H_2, 10 wt% LA in dioxane, and a substrate/metal weight ratio of 2000.

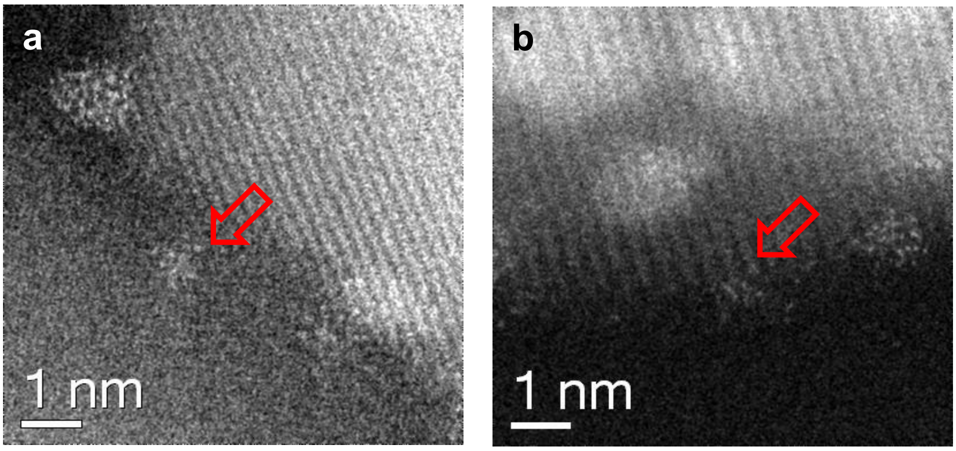

Supplementary Figure 9: Representative examples of high angle annular dark field (HAADF) images. (**a**) 1% Au-Pd/TiO_2 (M_{Im}). (**b**) 1% Ru-Pd/TiO_2 (M_{Im}). The selected images confirm the presence of sub-nm metal clusters.

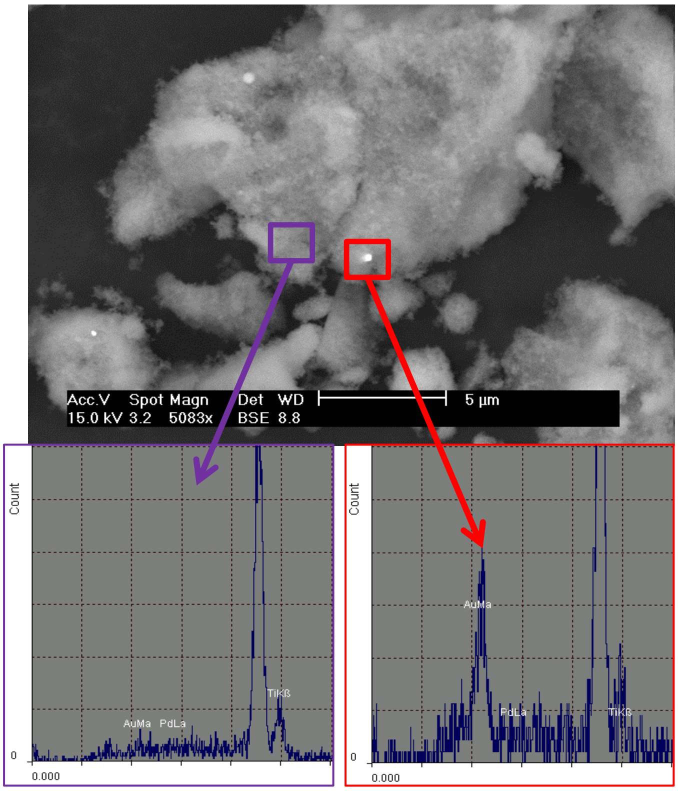

Supplementary Figure 10: A representative example of a SEM backscattered electron image of the 1% Au-Pd/TiO_2 (M_{Im}). The images show evidence for metal particles in the 0.1-0.5 µm size range. XEDS analysis of such particles confirmed them to be Au.

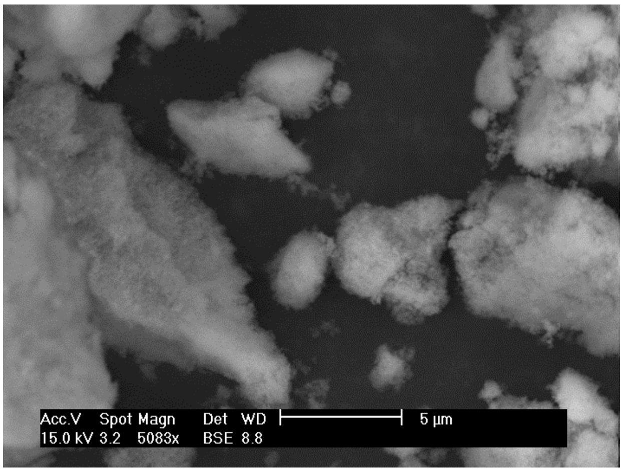

Supplementary Figure 11: A representative example of a SEM backscattered electron image of 1% Ru-Pd/TiO_2 (MIm). The image confirms that no micron-scale metal particles were formed.

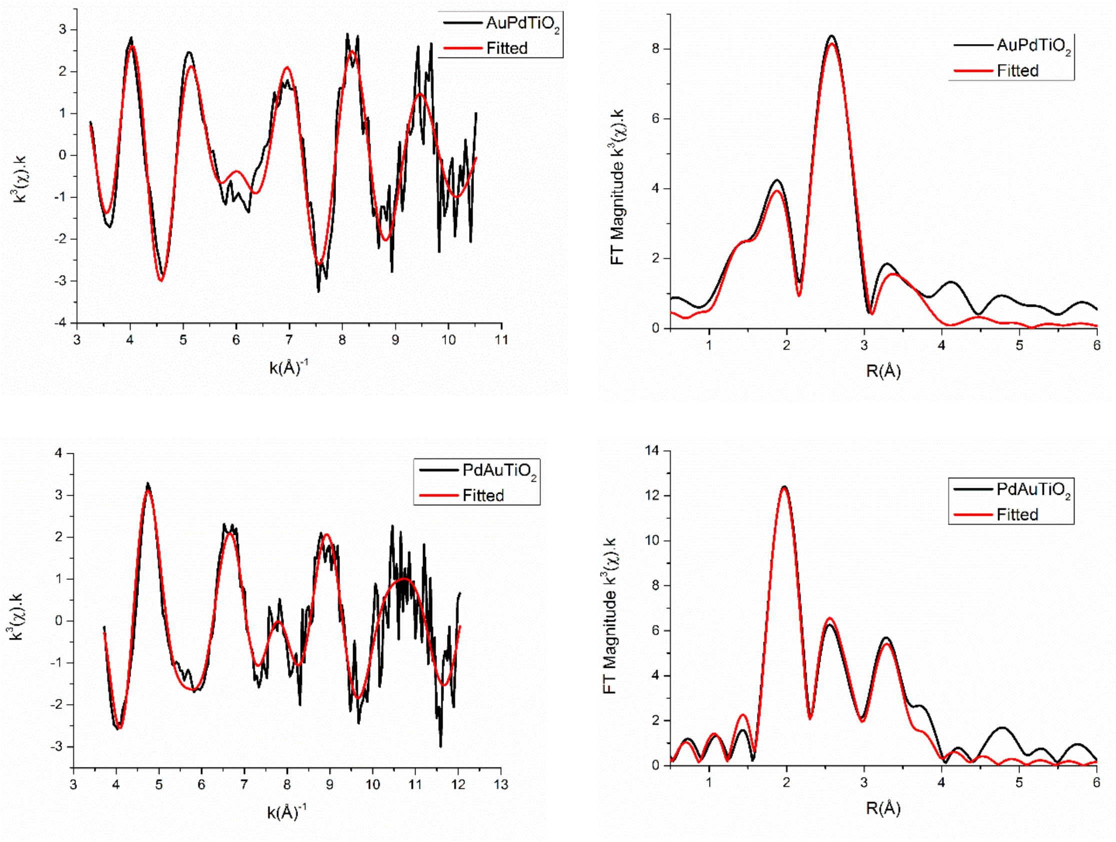

Supporting Figure 12: Isolated Au L_3 and Pd K-edge EXAFS and associated Fourier Transform data for the two-shell fits for 1% Au-Pd/TiO_2 (M_{Im}). Black line: data, red line: fit. EXAFS spectra were fitted in k-space.

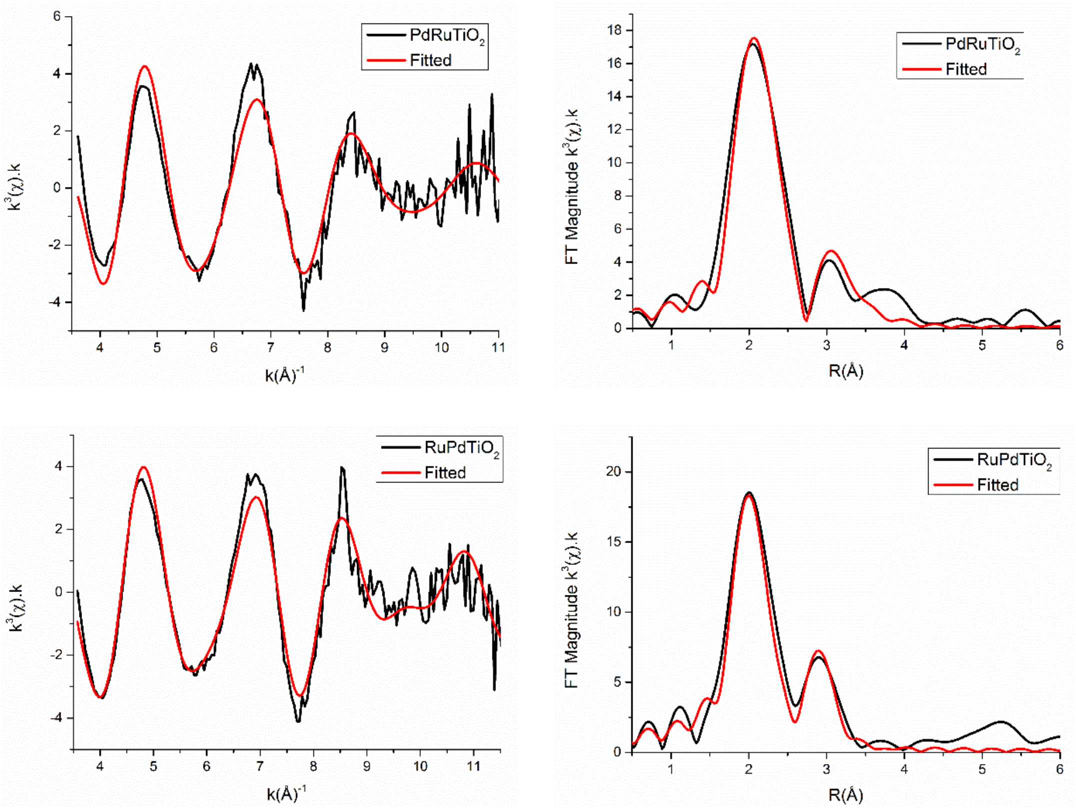

Supporting Figure 13: Isolated Pd and Ru K-edge EXAFS and associated Fourier Transform data for the two-shell fits for 1% Pd-Ru/TiO_2 (M_{Im}). Black line: data, red line: fit. EXAFS spectra were fitted in k-space.

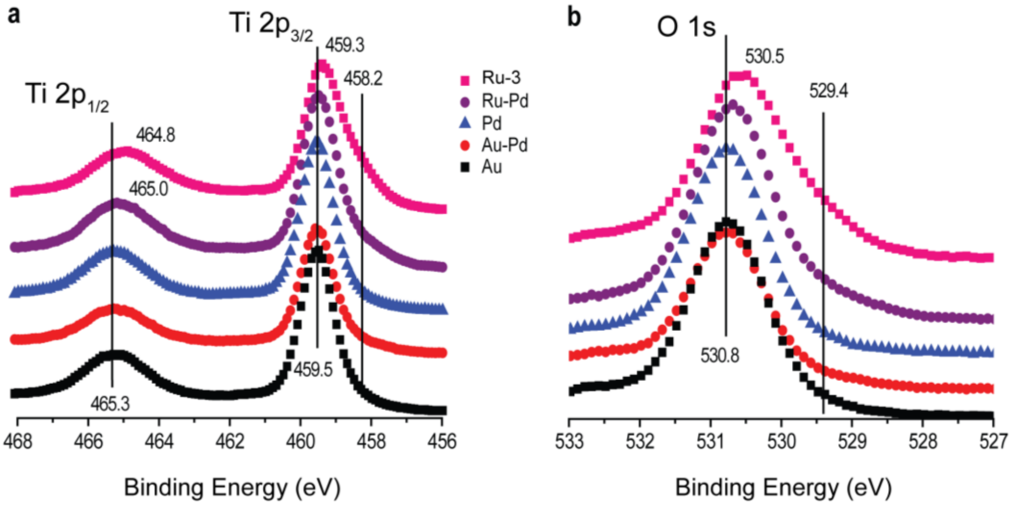

Supplementary Figure 14: X-ray photoelectron spectroscopy (XPS) data of the Ti 2p and O 1s regions. (**a**) Ti 2p region of various TiO_2-supported monometallic (Ru (0 M HCl), Pd and Au) and bimetallic (Au-Pd and Ru-Pd) catalysts. (**b**) O 1s region of various TiO_2-supported monometallic (Ru (0 M HCl), Pd and Au) and bimetallic (Au-Pd and Ru-Pd) catalysts.

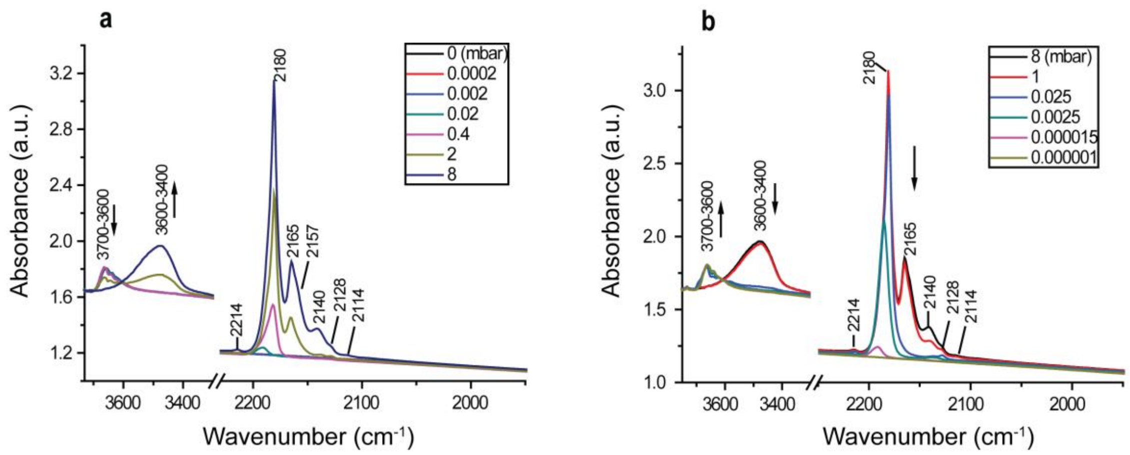

Supplementary Figure 15: FT-IR spectra of adsorbed CO on 1% Au/TiO_2 (M_{Im}). (**a**) Spectra measured during stepwise adsorption at 87 K. (**b**) Spectra measured during stepwise desorption under reduced pressure at 87 K. Mainly weak CO interactions with the support surface were observed on 1% Au/TiO_2 (M_{Im}), and only a marginal amount of Au-CO(L) at 2114 cm^{-1} is observed at 8 mbar CO pressure.

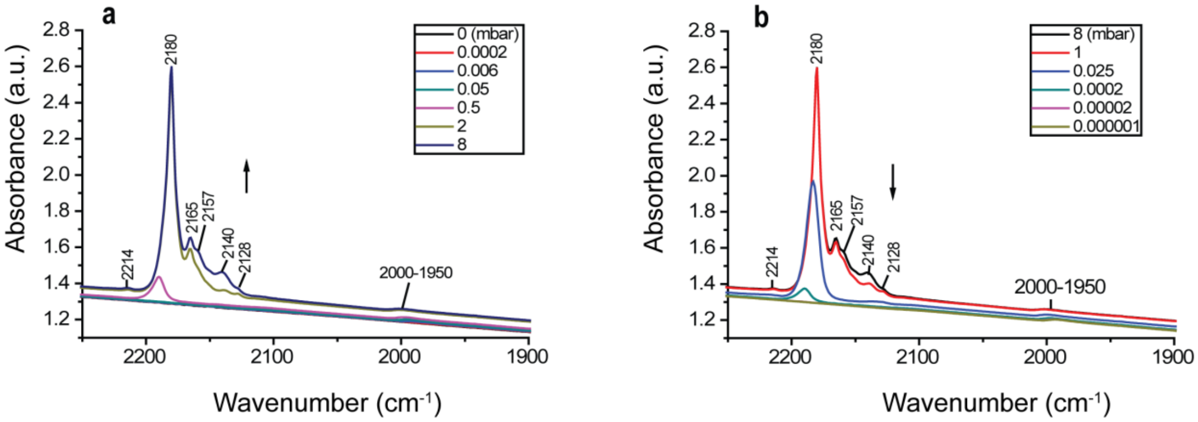

Supplementary Figure 16: FT-IR spectra of adsorbed CO on a 1% Pd/TiO_2 (M_{Im}) catalyst. (**a**) Spectra measured during stepwise adsorption at 87 K. (**b**) Spectra measured during stepwise desorption under reduced pressure at 87 K. Most features for 1% Pd/TiO_2 (M_{Im}) arise from weakly adsorbed CO on the support. The weak broad feature at 2000-1950 cm^{-1}, that remains after evacuation, is assigned to trace amounts of bridging carbonyl species on Pd/TiO_2 (M_{Im}).[1-3]

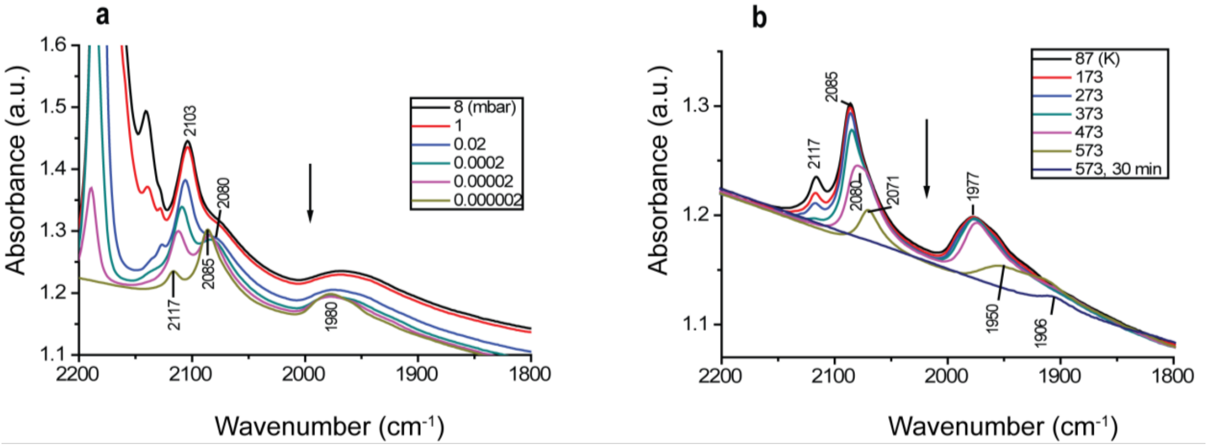

Supplementary Figure 17: FT-IR spectra of CO adsorbed on 1% Au-Pd/TiO_2 (M_{Im}). (**a**) Spectra measured during stepwise desorption from 8 to 10^{-6} mbar at 87 K. (**b**) Spectra measured during CO temperature-programmed desorption at 10^{-6} mbar. The IR bands of CO adsorbed on Pd can be distinguished into a linear mode Pd-CO(L) (2110–2050 cm^{-1}), two-fold bridged mode $Pd^{\delta+}$-CO(B) (1995-1975 cm^{-1}), Pd^0-CO(B) (1960–1925 cm^{-1}) and tri-fold bridged (1910–1870 cm^{-1}) modes;[4-6] CO adsorbed on Au only shows one linear Au-CO(L) (2120-2110 cm^{-1}) mode. Absence of signals between 2200 and 2125 cm^{-1} at $p_{CO} < 0.002$ mbar, suggests that Au does not bear a positive charge.

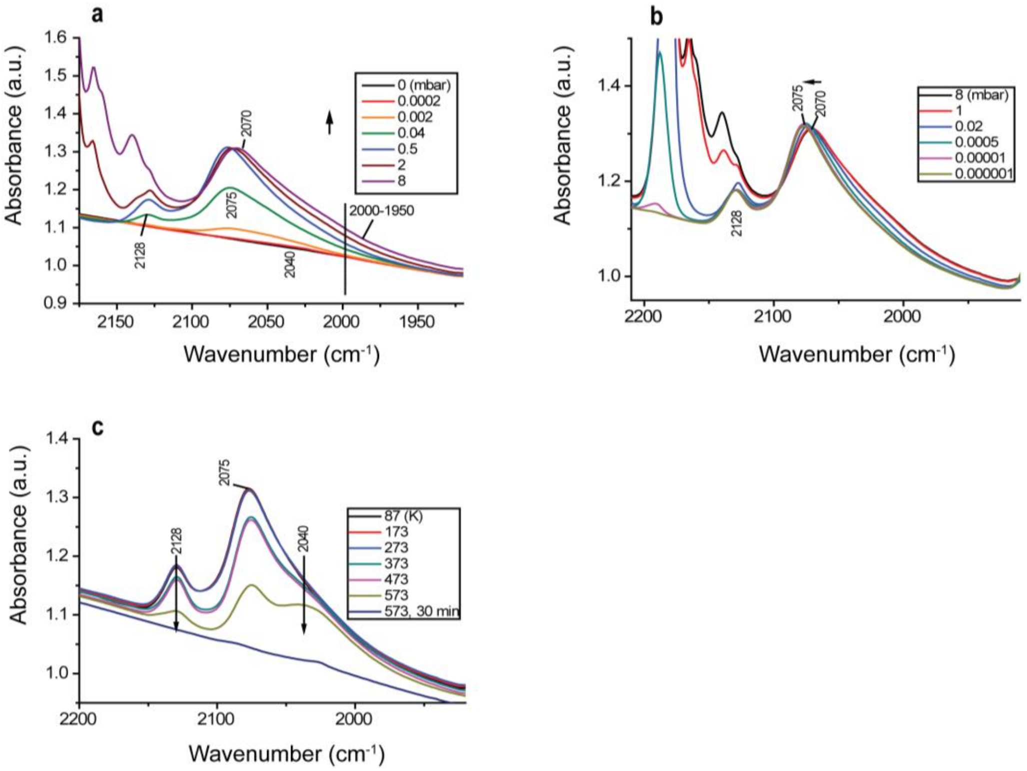

Supplementary Figure 18: FT-IR spectra of CO adsorbed on 1wt% Ru/TiO_2 (M_{Im}, 0 M HCl). (**a**) Spectra measured after stepwise adsorption from 10^{-6} to 8 mbar at 87 K. (**b**) Spectra measured during stepwise desorption from 8 to 10^{-6} mbar at 87 K. (**c**) Spectra measured during CO temperature programmed desorption at 10^{-6} mbar. At a low CO pressure of 0.002 mbar, a feature at 2075 cm^{-1} with a shoulder at 2040 cm^{-1} developed, which could be assigned to $Ru^{\delta+}$-CO (L), and Ru^{0}-CO (L) species, respectively.[7-10] In addition, a broad peak was seen centered ~1880 cm^{-1} for all the Ru-containing samples as a result of Ru^{4+}=O overtone species.[10,11] With further increase of the CO pressure to 0.04 mbar, a band at 2128 cm^{-1} and a low-frequency 'tail' at *ca.* 2000-1950 cm^{-1} appeared simultaneously, attributed to two differently adsorbed CO species on oxidized Ru species, multi-coordinated Ru^{n+}-$(CO)_x$ ($x > 2$) (L) and Ru^{n+}-(CO) (L) respectively.[8,12,13] In particular, the $Ru^{\delta+}$-CO(L) band at 2075 cm^{-1} has a higher intensity than the Ru^{0}-CO(L) band at 2040 cm^{-1} at 87 K. This together with the evidence for a shorter distance of Ru-O species of 1.96 Å in XAFS, and the electron transfer from Ru to TiO_2 seen by XPS, suggests a strong support metal interaction.

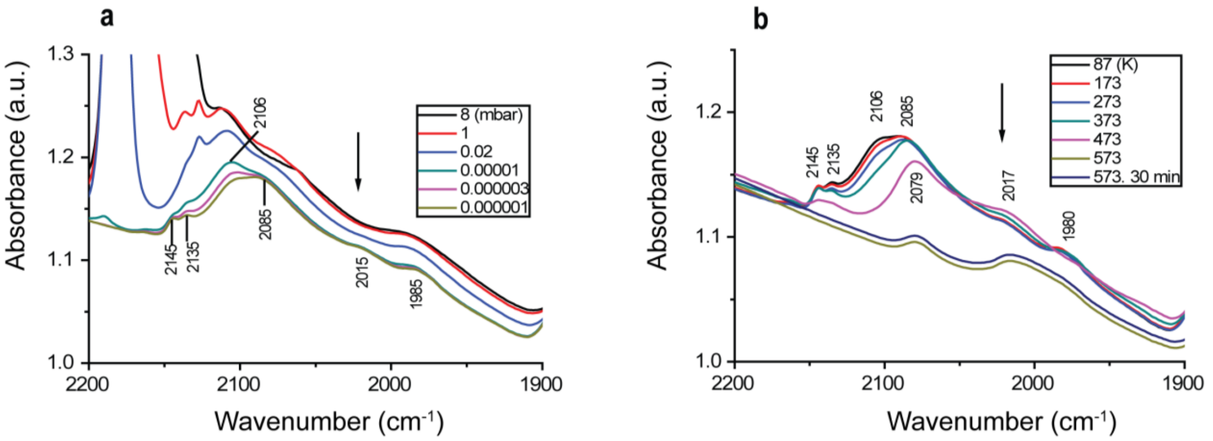

Supplementary Figure 19: FT-IR spectra of CO adsorbed on Ru-Pd/TiO_2 (M_{Im}). (**a**) Spectra measured during stepwise desorption from 8 to 10^{-6} mbar at 87 K. (**b**) Spectra measured during CO temperature programmed desorption at 10^{-6} mbar. The bands at 2150-2085 cm^{-1} can be attributed to Pd^{2+}-CO (L) species,[2,4] indicating the presence of oxidized Pd species.

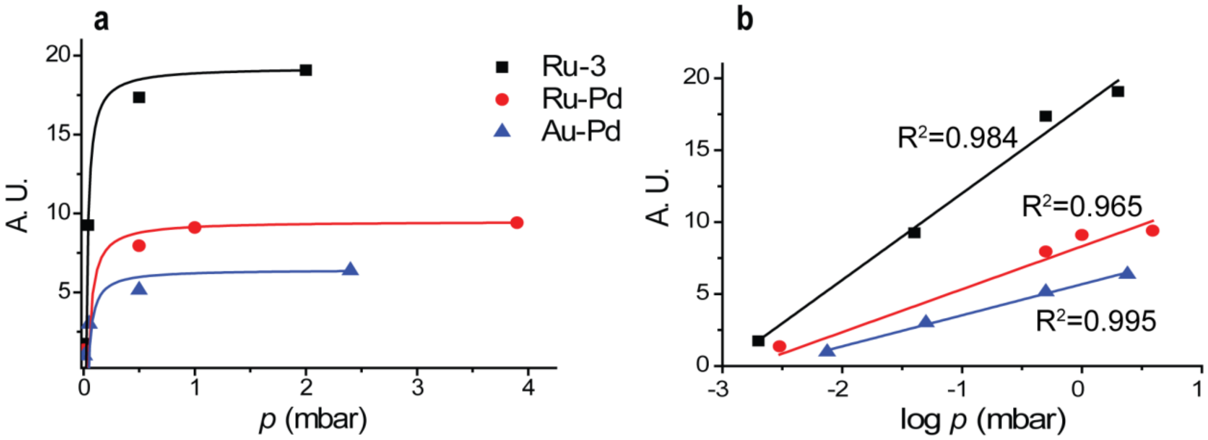

Supplementary Figure 20: CO adsorption plots as measured with FT-IR spectroscopy and related quantitative analysis of the CO peak intensity for the 1% Ru/TiO_2 (M_{Im}, 0 M HCl) (black squares), Au-Pd/TiO_2 (M_{Im}) (blue triangles) and 1% Ru-Pd/TiO_2 (M_{Im}) (red circles) catalysts. (**a**) Plot of the adsorbed linear CO peak intensity as a function of different p_{CO} values. (**b**) Adsorbed linear CO peak intensity for the metallic region versus logarithm of p_{CO}.

Supplementary Tables

Catalyst	TOF (s^{-1})	Reaction conditions	Reference
1% Ru/TiO_2 (M_{Im} 0 HCl)	1.949	473 K, 40 bar, in dioxane	Current work
1% Ru/TiO_2 (M_{Im})	0.536	473 K, 40 bar, in dioxane	Current work
1% Au/TiO_2 (M_{Im})	0.004	473 K, 40 bar, in dioxane	Current work
1% Pd/TiO_2 (M_{Im})	0.005	473 K, 40 bar, in dioxane	Current work
1% $AuPd/TiO_2$ (M_{Im})	0.102	473 K, 40 bar, in dioxane	Current work
1% $RuPd/TiO_2$ (M_{Im})	0.638	473 K, 40 bar, in dioxane	Current work
1% Ru/TiO_2 (W_{Im})[a]	0.239	473 K, 40 bar, neat LA	15
5% Ru/C	0.293	463 K, 12 bar, neat LA	16
5% Ru/C	0.033	403 K, 12 bar, ethanol/ water	16
5% Ru/TiO_2 (P25)	0.027	403 K, 12 bar, ethanol/ water	16
5% Ru/TiO_2	0.0059	423 K, 35 bar, in water	17
0.6% Ru/TiO_2	0.0137	423 K, 35 bar, in water	17
5% Pd/C	0.002	538 K, 1 bar, in dioxane	18
5% Ru/C+ Amberlyst-70	0.155	343 K, 30 bar, in water	19
RuSn(3.6: 1)/C	0.008	493 K, LA and FA in alkyl-phenol solvent	20
15% RuRe(3:4)/C	0.00049	423 K, 5 bar, equimolar LA and FA, and H_2SO_4 (0.5 mol/L)	21
1 mol% Au/ZrO_2	0.189	423 K, 5 bar, equimolar LA and FA	22
0.3wt% Ru0.9Ni0.1/ mesoporous carbon	0.606	423 K, 45 bar, LA in water	23

Supplementary Table 1: Productivity of different catalysts in the hydrogenation of LA to GVL, adapted from reference 14. [a] The 1% Ru/TiO_2 (W_{Im}) catalyst was prepared by a conventional wet impregnation method from its nitrate precursor.[15]

	**Ru loss** (%)/ppm	**Pd loss** (%)/ppm	**Au loss** (%)/ppm
1%Ru-Pd/TiO_2	1.0/0.5	0.4/0.2	
1%Au-Pd/TiO_2		1.1/0.4	0.5/0.3

Supplementary Table 2: Metal leaching into the liquid phase after reaction in dioxane as determined by Atomic Absorption Spectroscopy (AAS).

**Catalyst**		**Time**	**BET (m^2/g)**	**Pore volume (cm^3/g)**	**Coke content (wt%)**
1% Ru-Pd/TiO_2	Fresh		52.4	0.35	
	Spent	2 h	52.3	0.34	
	Spent	2 h $\times$ 3	48.0	0.35	2.3
1% Au-Pd/TiO_2	Fresh		51.5	0.35	
	Spent	4 h	47.0	0.35	
	Spent	4 h $\times$ 3	50.2	0.32	2.3

Supplementary Table 3: N_2 physisorption data of the fresh and spent bimetallic M_{Im} catalysts under investigation.

**Catalyst (M_{Im})**	**Binding Energy**							
	**Ti $2p_{1/2}$**	**Ti $2p_{3/2}$**	**Ti $2p_{3/2}$**	**O 1s**	**O 1s**	**Au $4f_{7/2}$**	**Pd $3d_{3/2}$**	**Ru $3d_{5/2}$**
1% Ru/TiO_2 (0 M HCl)	464.8	459.3	458.2	530.5	529.4			280.9
1% Ru-Pd/TiO_2	465.0	459.5	458.2	530.7	529.4		341.6	280.6
1% Pd/TiO_2	465.3	459.5		530.8			340.6	
1% Au-Pd/TiO_2	465.3	459.5		530.8		84.0	340.9	
1% Au/TiO_2	465.3	459.5		530.8		84.2		

Supplementary Table 4: Measured XPS binding energies (BE) for different M_{Im} catalysts. Due to overlap of the Au $4f_{5/2}$ and Pd 4s peaks, only the Au $4f_{7/2}$ signal could be used to assess the electronic properties of Au; the Pd $3d_{3/2}$ signals were used to study the electronic properties of Pd, as the Pd $3d_{5/2}$ and Au $4d_{5/2}$ peaks overlapped. The C 1s signal (an artefact from the sample holder) was used as an internal reference.

Supplementary References:

1.. Kuhn, W. K., Szanyi, J. & Goodman, D. W. CO adsorption on Pd(111): the effects of temperature and pressure. *Surf. Sci.* **274**, L611-L618 (1992).

2. Li, Y. *et al.* The effect of titania polymorph on the strong metal-support interaction of Pd/TiO_2 catalysts and their application in the liquid phase selective hydrogenation of long chain alkadienes. *J. Mol. Catal. A: Chemical* **216**, 107-114 (2004).

3. Ozensoy, E. & Goodman, D. W. Vibrational spectroscopic studies on CO adsorption, NO adsorption CO plus NO reaction on Pd model catalysts. *Phys. Chem. Chem. Phys* **6**, 3765-3778 (2004).

4. Tessier, D., Rakai, A. & Bozon-Verduraz, F. Spectroscopic study of the interaction of CO with cationic and metallic Pd in Pd-alumina catalysts. *J. Chem. Soc. Faraday Trans.* **88**, 741-749 (1992).

5. Kolli, N. E., Delannoy, L. & Louis, C. Bimetallic Au–Pd catalysts for selective hydrogenation of butadiene: Influence of the preparation method on catalytic properties. *J. Catal.* **297**, 79-92 (2013).

6. Wei, T., Wang, J. & Goodman, D. W. Characterization and chemical properties of Pd−Au alloy surfaces. *J. Phys. Chem. C* **111**, 8781-8788 (2007).

7. Robbins, J. L. Chemistry of supported Ru: CO-induced oxidation of Ru at 310 K. *J. Catal.* **115**, 120-131 (1989).

8. Yokomizo, G. H., Louis, C. & Bell, A. T., An infrared study of CO adsorption on reduced and oxidized Ru/SiO_2. *J. Catal.* **120**, 1-14 (1989).

9. Guglielminotti, E. & Bond, G. C. Effect of oxidation-reduction treatments on the infrared spectra of CO chemisorbed on a Ru/TiO_2 catalyst. *J. Chem. Soc. Faraday Trans.* **86**, 979-987 (1990).

10. Hadjiivanov, K. *et al.* FTIR Study of CO Interaction with Ru/TiO_2 Catalysts. *J. Catal.* **176**, 415-425 (1998).

11. Lopez, T., Bosch, P., Asomoza, M. & Gomez, R. Ru/SiO_2-impregnated and sol-gel-prepared catalysts: synthesis, characterization, and catalytic properties. *J. Catal.* **133**, 247-259 (1992).

12. Gupta, N. M. *et al.* The transient species formed over RuO_x/TiO_2 catalyst in the CO and CO + H_2 interaction: FT-IR spectroscopic study. *J. Catal.* **137**, 473-486 (1992).

13. Elmasides, C., Kondarides, D. I., Neophytides, S. G. & Verykios, X. E. *Stud. Surf. Sci. Catal.* **130**, 3083-3088 (2000).

14. Wright, W. R. H. & Palkovits, R. Development of heterogeneous catalysts for the conversion of levulinic acid to γ-valerolactone. *ChemSusChem* **5**, 1657-1667 (2012).

15. Luo, W. *et al.* Ruthenium-catalyzed hydrogenation of levulinic acid: Influence of the support and solvent on catalyst selectivity and stability. *J. Catal.* **301**, 175-186 (2013).

16. Al-Shaal, M. G., Wright, W. R. H. & Palkovits, R. Exploring the ruthenium catalysed synthesis of γ-valerolactone in alcohols and utilisation of mild solvent-free reaction conditions. *Green Chem.* **14**, 1260-1263 (2012).

17. Primo, A., Concepcion, P. & Corma, A. Synergy between the metal nanoparticles and the support for the hydrogenation of functionalized carboxylic acids to diols on Ru/TiO_2. *Chem. Commun.* **47**, 3613-3615 (2011).

18. Upare, P. P. *et al.* Selective hydrogenation of levulinic acid to γ-valerolactone over carbon-supported noble metal catalysts. *Ind. Eng. Chem.* **17**, 287-292 (2011).

19. Galletti, A. M. R., Antonetti, C., De Luise, V. & Martinelli, M., A sustainable process for the production of γ-valerolactone by hydrogenation of biomass-derived levulinic acid. *Green Chem.* **14**, 688-694 (2012).

20. Alonso, D. M. *et al.* Production of biofuels from cellulose and corn stover using alkylphenol solvents. *ChemSusChem* **4**, 1078-1081 (2011).

21. Braden, D. J. *et al.* Production of liquid hydrocarbon fuels by catalytic conversion of biomass-derived levulinic acid. *Green Chem.* **13**, 1755-1765 (2011).

22. Du, X.-L. *et al.* Hydrogen-independent reductive transformation of carbohydrate biomass into γ-valerolactone and pyrrolidone derivatives with supported gold catalysts. *Angew. Chem. Int. Ed.* **123**, 7961-7965 (2011).

23. Yang, Y., Gao, G., Zhang, X. & Li, F., Facile fabrication of composition-tuned Ru–Ni bimetallics in ordered mesoporous carbon for levulinic acid hydrogenation. *ACS Catal.* **4**, 1419–1425 (2014).
